# Altered endothelial mitochondrial Opa1‐related fusion in mouse accelerates age‐associated vascular and kidney damage

**DOI:** 10.14814/phy2.70451

**Published:** 2025-07-04

**Authors:** Carlotta Turnaturi, Loïck L'Hoste, Coralyne Proux, Linda Grimaud, Emilie Vessieres, Antonio Zorzano, Anne Teissier, Pascal Reynier, Raffaella Sorrentino, Guy Lenaers, Laurent Loufrani, Daniel Henrion

**Affiliations:** ^1^ Univ Angers, INSERM, CNRS, MITOVASC Dept, CARME Team, SFR ICAT Angers France; ^2^ Department of Pharmacy, School of Medicine and Surgery University of Naples Federico II Naples Italy; ^3^ Department of Cardiac Surgery University Hospital (CHU) of Angers Angers France; ^4^ Institute for Research in Biomedicine (IRB Barcelona) The Barcelona Institute of Science and Technology Barcelona Spain; ^5^ Departament de Bioquímica i Biomedicina Molecular, Facultat de Biologia Universitat de Barcelona Barcelona Spain; ^6^ Centro de Investigación Biomédica en Red de Diabetes y Enfermedades Metabólicas Asociadas (CIBERDEM) Instituto de Salud Carlos III Madrid Spain; ^7^ Department of Biochemistry and Molecular Biology University Hospital of Angers Angers France; ^8^ Department of Neurology University Hospital of Angers Angers France

**Keywords:** aging, arteries, endothelial cell, kidney, mitochondrial fusion

## Abstract

Cardiovascular diseases are the major cause of death worldwide, and their frequency increases with age in association with kidney damage. As a reduction in fusion protein optic atrophy type 1 (Opa1) level in endothelial cells (ECs) decreases the vascular response to flow and increases oxidative stress in perfused kidneys, we hypothesized that reduced Opa1 expression contributes to vascular aging. We used male and female mice with ECs specific Opa1 knock‐out (EC‐Opa1), and littermate wild‐type (EC‐WT) mice aged 6 (young) and 20 months (old). Mesenteric resistance arteries (MRA) and kidneys were collected for vascular reactivity and western‐blot analysis. In old EC‐Opa1 mice, blood urea was greater than in EC‐WT mice, and MRA showed reduced endothelium‐dependent relaxation. In kidneys, the mitochondria fission protein fission‐1 (Fis‐1) and the peroxisome proliferator‐activated receptor gamma coactivator‐1 alpha (Pgc‐1α) were increased in old EC‐Opa1 mice. The level of caveolin‐1 expression was greater in old EC‐Opa1 mice. Moreover, in kidneys from EC‐Opa1 old mice, NADPH‐oxidase subunit gp91 expression was greater than in age‐matched EC‐WT mice. Thus, reduced mitochondrial fusion in mouse ECs altered mesenteric vascular reactivity and increased markers of oxidative stress in aging kidneys. Thus, Opa1 might protect the vascular tree in target organs such as the kidney.

## Graphical abstract

Mitochondria fusion protects endothelial cells in aging through a reduction in ROS production by NOX2 and through a reduction of the inhibitory effect of Cav‐1 on eNOS, thus allowing NO production and vascular relaxation.

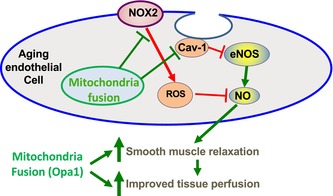

## 1 INTRODUCTION

Aging is the most important risk factor for cardiovascular disorders (Donato et al., 2018; Lakatta & Levy, 2003). The endothelium has a central role in vascular homeostasis, and endothelial cells (ECs) are the front‐line cells against vascular diseases (Xu et al., 2022) explaining why dysregulation of vascular endothelial cells is a major cause of cardiovascular disease (CVD) such as atherosclerosis, arterial hypertension, coronary artery disease, ischemia reperfusion injury, and myocarditis (Favero et al., 2014). The kidney has a dense microvascular network and is an early target organ in CVD. Renal ECs are essential for kidney health, as they control vascular integrity and regulate blood flow, thus allowing renal filtration. During aging, renal ECs become less efficient, leading to kidney dysfunction, as detailed in a recent review (Borri et al., 2025).

Blood flow generates shear stress at the intimal surface of ECs, inducing a permanent vasodilator tone due to flow (shear stress)‐mediated dilation (FMD). FMD reduces vascular resistance and thus improves renal filtration. Interestingly, in humans, FMD can be measured non‐invasively in the forearm (Joannides et al., 1995; Zhou et al., 2014) and a decreased FMD is a hallmark of endothelium dysfunction (Zhou et al., 2014).

ECs have a small number of mitochondria, and their adenosine‐5′triphosphate (ATP) production relies on glycolysis more than on mitochondria (Culic et al., 1997). Nevertheless, mitochondria play a major role in the regulation of ECs function (Kadlec et al., 2016). They participate in cellular homeostasis and have a role in reactive oxygen species (ROS) production and in the regulation of Ca^2+^ concentration in the cytosol of ECs (Tang et al., 2014). Furthermore, mitochondrial ATP is essential for calcium‐dependent, nitric oxide‐mediated vasodilation and thus for the control of vascular tone by the endothelium (Wilson et al., 2023). This study agrees with our previous work showing the key role of endothelial mitochondrial fusion in FMD (Chehaitly et al., 2022).

Mitochondria are dynamic organelles that undergo continuous cycles of fusion and fission. Mitochondrial fusion is mediated by mitofusin‐1 (Mfn1), mitofusin‐2 (Mfn2) and optic atrophy type 1 (OPA1), and fission is mediated by dynamin‐related protein 1 (DRP1) and mitochondrial fission‐1 protein (Fis1). Mfn1 and Mfn2 are responsible for the outer membrane fusion, while OPA1 is necessary for the inner membrane fusion. For fission, DRP1, a cytosolic protein, aggregates to Fis1 and is anchored to the outer membrane to promote mitochondrial membrane fission. A balanced equilibrium between fission and fusion is important for mitochondrial integrity and thus for mitochondrial energy production, calcium homeostasis, and adaptation to changing environments. OPA1 is also important for the maintenance of cristae structure of mitochondria and thus for better OXPHOS respiration (Frezza et al., 2006).

The formation of new mitochondria is regulated by the peroxisome proliferation‐activated receptor gamma co‐activator 1α (Pgc‐1α), which is a transcriptional activator of nuclear respiratory factor (Nrf)1. Mitochondrial biogenesis diminishes with age, leading to mitochondrial dysfunction (Fannin et al., 1999) with less energy production, increased ROS production, and oxidative damage (Giorgi et al., 2018). A loss of OPA1 leads to the disorganization of cristae and mitochondrial fragmentation associated with excessive ROS production (Cipolat et al., 2006; Kao et al., 2015; Tang et al., 2009; Yarosh et al., 2008). We have previously shown that *Opa1* ^+/−^ mice are more susceptible to arterial hypertension (Robert et al., 2021) and that FMD is selectively reduced in a mouse model with Opa1 deficiency in ECs only (EC‐Opa1 mice) (Chehaitly et al., 2022). In this latter study, we showed that FMD was reduced in small resistance arteries, generating a deterioration of the flow‐pressure relationship in the perfused kidney in young EC‐Opa1 mice. This was associated with excessive ROS production in arteries and in the perfusate of the isolated kidney. No difference was observed between male and female mice (Chehaitly et al., 2022).

Thus, we hypothesized that the mitochondrial fusion deficiency in ECs accelerates vascular and kidney damage in aging. To address this question, we used the mouse model previously described with *Opa1* knock‐out in ECs (Chehaitly et al., 2022) and investigated vascular reactivity in mesenteric arteries and measured markers of oxidative stress in the kidney in male and female mice aged 6‐ and 20 months.

## 2 MATERIALS AND METHODS

### 2.1 Mice

As previously described (Chehaitly et al., 2022) mice (Mus Musculus Opa1 flox/flox X VECadh‐creER T2, MGI: 3848982) lacking Opa1 in ECs were obtained after crossing *Cadherin5‐CreERT2* mice with *Opa1* ^*loxP/loPx*^ mice (MGI: 4364874) (Rodriguez‐Nuevo et al., 2018). They were designed as EC‐Opa1 mice (*Cadherin5‐CreERT2* ^+^ *Opa1* ^*loxP/loPx*^) and were compared to their littermate control EC‐WT mice (*Cadherin5‐CreERT2* ^−^ *Opa1* ^*loxP/loxP*^). The deletion was induced by injection of tamoxifen (150 mg/kg per day, Merck Sigma Aldrich # T5648, CAS number: 10540‐29‐1), diluted in corn oil (Merck Sigma Aldrich # C8267‐500ML CAS number: 8001‐30‐7) during five consecutive days in mice aged 3 months. Mice were then used 3 (young mice) or 17 months (old mice) after tamoxifen induction. Thus, mice included in the study were aged 6 months (young mice, 44 animals used in the study) or 20 months (old mice, 40 animals used in the study). We did not use mice older than 20 months to avoid the occurrence of too severe organ dysfunctions that would make it difficult to see an effect of the reduced Opa1 expression in ECs.

Mice were housed under standard conditions (agreement numbers of the animal facility: L1‐0203 and A1‐0216), with a temperature of 23 ± 1°C, 12‐h light/dark cycle, and free access to dry food (Safe® A04) and water.

Male and female mice were used in the study. In a previous study using EC‐Opa1and EC‐WT mice, we have not observed significant differences between male and female mice (Chehaitly et al., 2022).

All procedures were performed in accordance with the principles and guidelines established by the National Institute of Medical Research (INSERM) and were approved by the local Animal Care and Use Committee (APAFIS#2018011217209, APAFIS#30385‐2021031010145750). The investigation conforms to the directive 2010/63/EU of the European Parliament.

### 2.2 Vascular reactivity in mesenteric arteries in vitro

Segments of first order mesenteric arteries were carefully dissected free of fat and connective tissues. They were then mounted in a 610 M wire‐myograph (Danish MyoTechnology, DK) as previously described (Guivarc'h et al., 2020). Briefly, two tungsten wires were inserted into a 2 mm long arterial segment; one was fixed to a force transducer and one to a micrometer. They were continuously bathed in a physiological salt solution (PSS) of the following composition (mM): 130, NaCl; 15, NaHCO_3_; 3.7, KCl; 1.2, KH_2_PO_4_; 1.2, MgSO_4_; 11, glucose; 1.6, CaCl_2_; and 5, HEPES, pH 7.4, pO_2_ 160 mmHg, pCO_2_ 37 mmHg. Wall tension was applied as described previously (Mulvany & Halpern, 1977). Arterial contractility was tested using phenylephrine (10^−9^ to 3.10^−5^ mol/L, Merck Sigma Aldrich # P6126‐5G CAS number: 61‐76‐7).). Endothelial function was then tested using acetylcholine (ACh, 10^−9^ to 3.10^−5^ mol/L, Merck Sigma Aldrich # A6625‐25G CAS number: 60‐31‐1) after precontraction with Phe (10^−6^ mol/LEndothelium‐independent relaxation was tested with sodium nitroprusside (SNP, 10^−9^ to 10^−5^ mol/L, Merck Sigma Aldrich # 71778‐25G CAS number: 13755‐38‐9) after precontraction with Phenylephrine (10^−6^ mol/L).

### 2.3 Analysis of protein expression levels by western blot

Kidney proteins were extracted using an extraction buffer of the following composition: SDS 0.1%, Tris 10 mM pH 7,4, proteases inhibitors 1X (CAT#78444, Thermo Fisher Scientific, Waltham, MA, USA), EDTA 0.5 mM. Homogenates were centrifuged at 13000 rpm at 4°C for 20 min, and the resulting supernatant was collected. Protein concentration was determined using the Micro BCA protein assay kit (cat#23227, Thermo Fisher Scientific, Waltham, MA, USA) according to the manufacturer's instructions. Equal amounts of proteins (30 μg) were solubilized in 25 μL of Laemmli sample buffer containing 2.5% β‐mercaptoethanol, boiled for 5 min at 90°C, separated by 4%–15% polyacrylamide gel electrophoresis (BioRad, Marnes la Coquette, France) and transferred to a nitrocellulose membrane (BioRad). Membranes were incubated overnight at 4°C with the primary antibody followed by the appropriate peroxidase‐labeled secondary antibody for 1 h. Reactions were visualized by ECL (Clarity Western ECL Substrate Bio‐Rad #1705061) detection according to the manufacturer's instructions (Bio‐Rad, Marnes‐la‐Coquette, France) and membranes were stripped at room temperature for 20 min twice in the presence of a low pH glycine solution before re‐blotting. The list of antibodies used in the present study is given in the (Table S1).

### 2.4 Blood urea nitrogen level in mice

Blood urea nitrogen was measured using the Atellica™ CH Urea Nitrogen UN‐c assay (reference # 11097593, Siemens, Erlangen, Germany).

### 2.5 Statistical analyses

For concentration‐response curves, a two‐way ANOVA for repeated measurements followed by a Bonferroni's post‐test was performed. For the other comparisons, two‐way ANOVA followed by a Bonferroni's post‐test was used, as indicated in the figure legends. Probability values lower than 0.05 were considered significant. Data from male and female mice were pooled.

## 3 RESULTS

### 3.1 Blood urea nitrogen measurements

**FIGURE 1:**
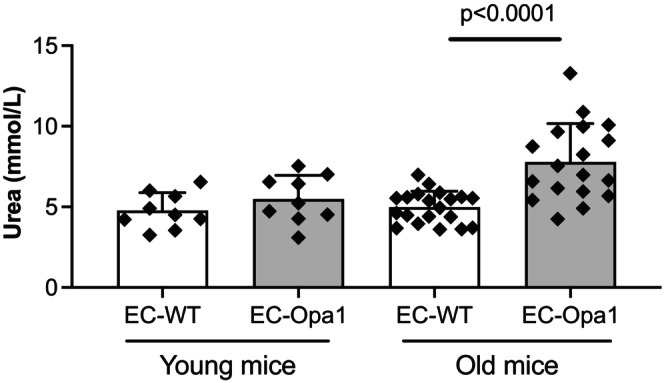
Blood urea nitrogen measurement. Blood urea nitrogen was measured in young and old EC‐Opa1 and EC‐WT mice. Data are expressed as means ± SD (*n* = 9 EC‐WT mice, 9 young EC‐Opa1 young mice, 20 old EC‐WT mice and 18 EC‐Opa1 old mice). Two‐way ANOVA and Bonferroni's multiple comparisons test.

Blood urea nitrogen was equivalent in young EC‐Opa1 and EC‐WT (Figure 1). It was not significantly different in old EC‐Opa1 mice compared to young EC‐WT mice (Figure 1). Nevertheless, blood urea nitrogen was significantly elevated in old EC‐Opa1 mice compared to old EC‐WT mice (Figure 1).

### 3.2 Vascular contractility in mesenteric arteries

**FIGURE 2:**
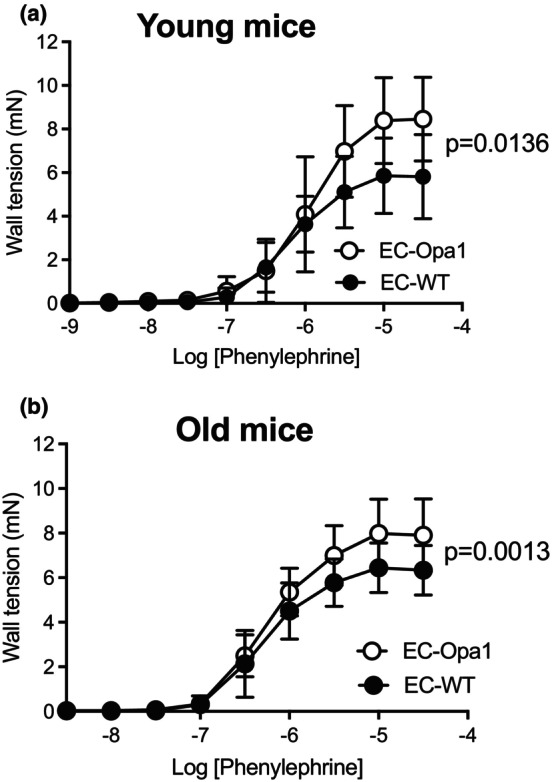
Phenylephrine‐mediated contraction. Phenylephrine (1 nmol/L to 30 μmol/L)‐mediated cumulative concentration‐response curve was determined in mesenteric arteries isolated from young (a) and old (b) EC‐Opa1 and EC‐WT mice. Data are expressed as mean ± SD (*n* = 12 young EC‐Opa1 mice, 13 young EC‐WT mice, 19 old EC‐Opa1 mice and 21 old EC‐WT mice). Two‐way ANOVA for repeated measurements and Bonferroni's multiple comparisons test.

Phenylephrine‐mediated contraction was measured in isolated mesenteric arteries. In both young and old mice, phenylephrine‐mediated contraction was significantly higher in EC‐Opa1 than in age‐matched EC‐WT mice (Figure 2a,b).

### 3.3 Endothelium‐dependent relaxation in mesenteric arteries

**FIGURE 3:**
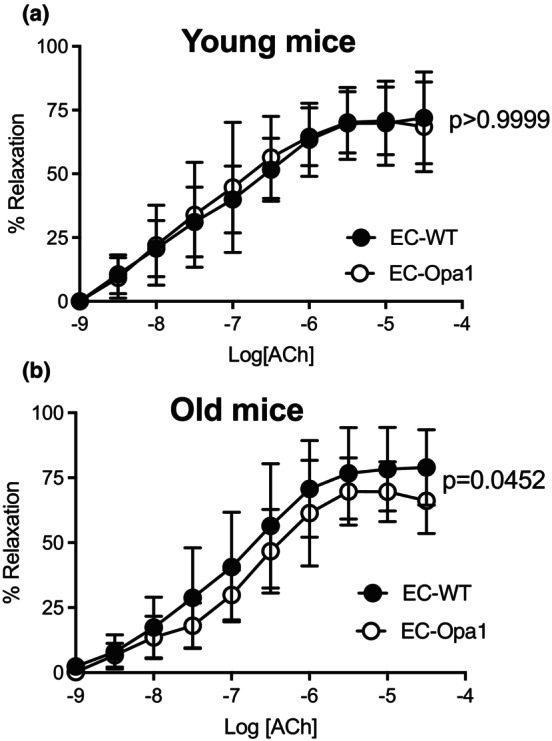
Acetylcholine‐mediated endothelium‐dependent relaxation. Acetylcholine (ACh, 1 nmol/L to 30 μmol/L)‐mediated endothelium‐dependent relaxation was determined in mesenteric arteries isolated from young (a) and old (b) EC‐Opa1 and EC‐WT mice. Data are expressed as means ± SD (*n* = 12 young EC‐Opa1 mice, 13 young EC‐WT mice, 19 old EC‐Opa1 mice and 21 old EC‐WT mice). Two‐way ANOVA for repeated measurements and Bonferroni's multiple comparisons test.

ACh induced endothelium‐dependent relaxation in mesenteric arteries (Figure 3).

In young mice, ACh‐mediated relaxation was equivalent in EC‐Opa1 and EC‐WT mice (Figure 3a) while in old mice, a significant reduction in relaxation was observed in EC‐Opa1 mice compared to EC‐WT mice (Figure 3b).

### 3.4 Endothelium‐independent relaxation in mesenteric arteries

**FIGURE 4:**
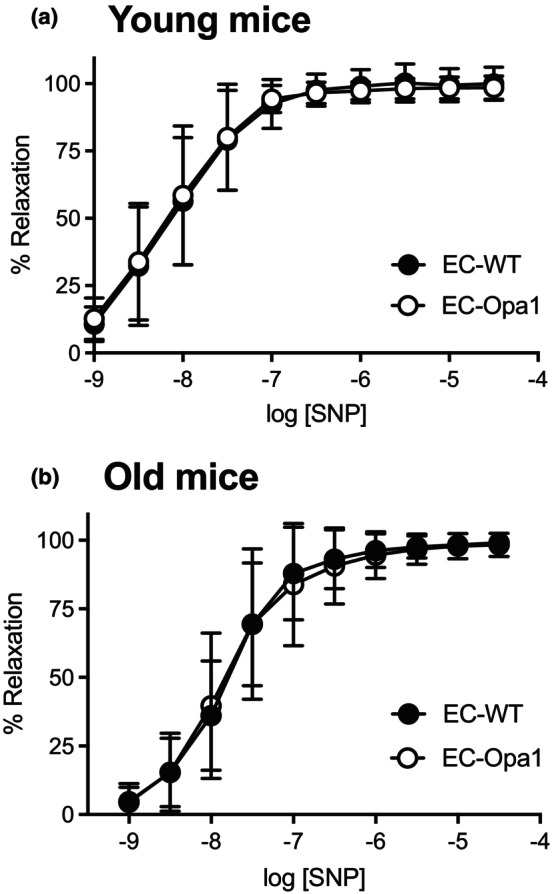
Sodium nitroprusside‐mediated endothelium‐independent relaxation. Sodium nitroprusside (SNP, 1 nmol/L to 30 μmol/L)‐mediated endothelium‐dependent relaxation was determined in mesenteric arteries isolated from young (a) and old (b) EC‐Opa1 and EC‐WT mice. Data are expressed as means ± SD (*n* = 12 young EC‐Opa1 mice, 13 young EC‐WT mice, 19 old EC‐Opa1 mice and 21 old EC‐WT mice). NS, Two‐way ANOVA for repeated measurements and Bonferroni's multiple comparisons test.

SNP induced endothelium‐independent relaxation in isolated mesenteric arteries (Figure 4). We observed no significant difference between groups.

### 3.5 Analysis of protein expression in kidneys

**FIGURE 5:**
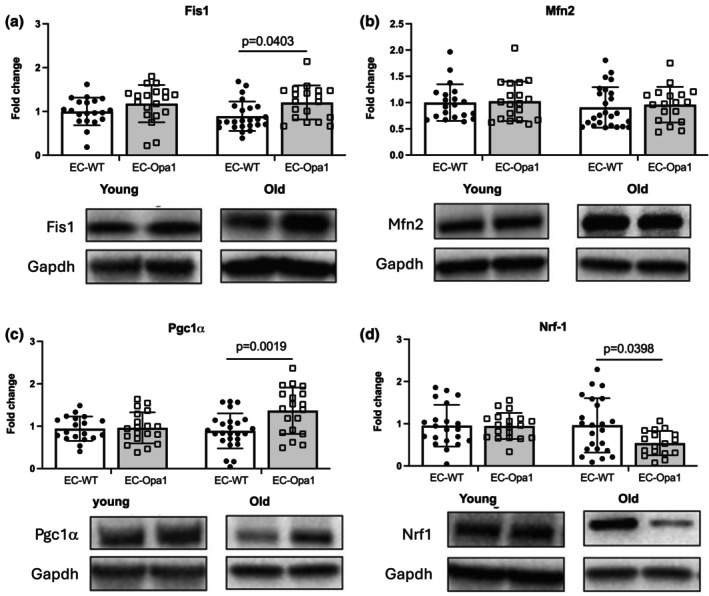
Protein expression level of fission‐1 protein (Fis1), proliferation‐activated receptor gamma co‐activator 1α (Pgc1α), mitofusin‐2 (Mfn2) and nuclear respiratory factor (Nrf‐1) in the kidney. Protein expression level of Fis1 (a), Mfn2 (b), Pgc1α (c) and Nrf‐1 (d) was determined in kidneys isolated from young and old EC‐WT and EC‐Opa1. Mean ± SD is shown (*n* = 21–27 mice per group). Young and old samples came from separate blots. Complete blot panels with marker proteins can be viewed in the Figure S8. Two‐way ANOVA and Bonferroni's multiple comparisons test.

The expression level of the mitochondrial fission protein Fis1 was significantly increased in the kidney in old EC‐Opa1 mice compared to old EC‐WT mice (Figure 5a) while the expression level of the mitochondrial fusion protein Mfn2 was not affected (Figure 5b). The expression level of Fis‐1 and Mfn2 was not affected by the absence of Opa1 in the endothelium in young mice (Figure 5a,b).

The expression level of Pgc‐1α, which promotes mitochondrial biogenesis, was significantly increased in old EC‐Opa1 mice compared to old EC‐WT mice (Figure 5c), while no difference was observed in young mice. Surprisingly, Nrf‐1 expression level, which is transcriptionally activated by Pgc‐1α, was significantly decreased in EC‐Opa1 old mice compared to EC‐WT old mice (Figure 5d).

**FIGURE 6:**
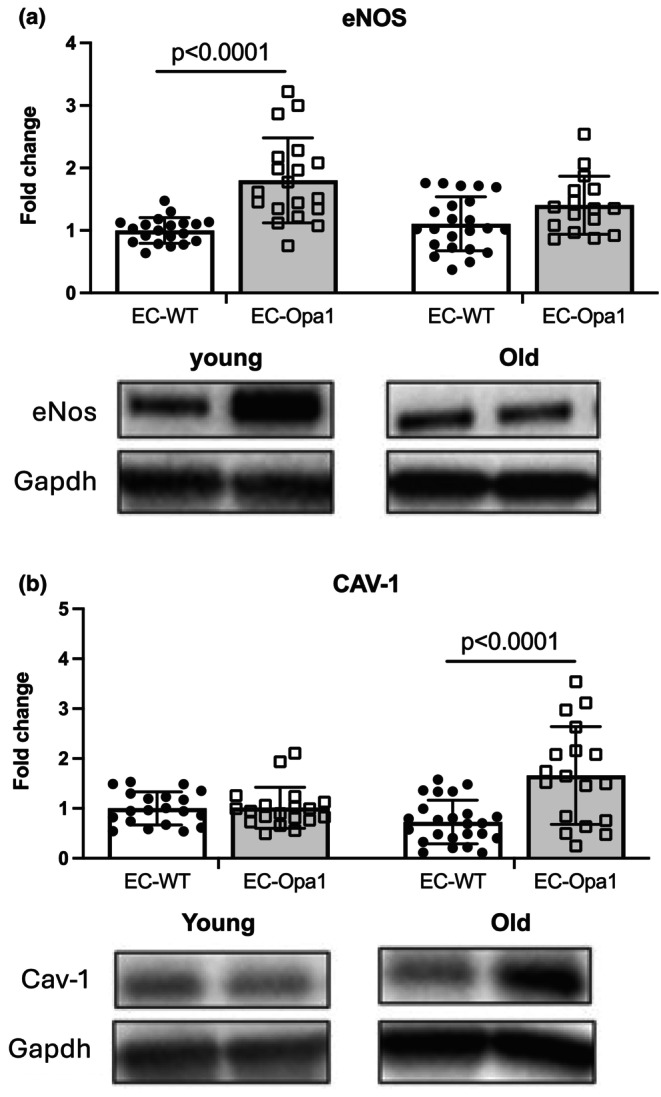
Protein expression level of endothelial nitric oxide enzyme (eNos) and Caveoline‐1 (Cav‐1) in the kidney. Protein expression level of eNos (a) and Cav‐1 (b) was determined in kidneys isolated from young and old EC‐WT and EC‐Opa1 mice. Mean ± SD is shown (*n* = 21–27 mice per group). Young and old samples came from separate blots. Complete blot panels with marker proteins can be viewed in the Figure S8. Two‐way ANOVA and Bonferroni's multiple comparisons test.

The expression level of endothelial nitric oxide (eNos) enzyme in kidneys was significantly increased in young EC‐Opa1 mice compared to young EC‐WT mice (Figure 6a) whereas no significant change in eNos expression level was observed in old EC‐Opa1 mice compared to old EC‐WT mice.

By contrast, caveolin‐1 (Cav‐1) expression level was significantly increased in old EC‐Opa1 mice compared to old EC‐WT mice without significant change in young mice (Figure 6b).

**FIGURE 7:**
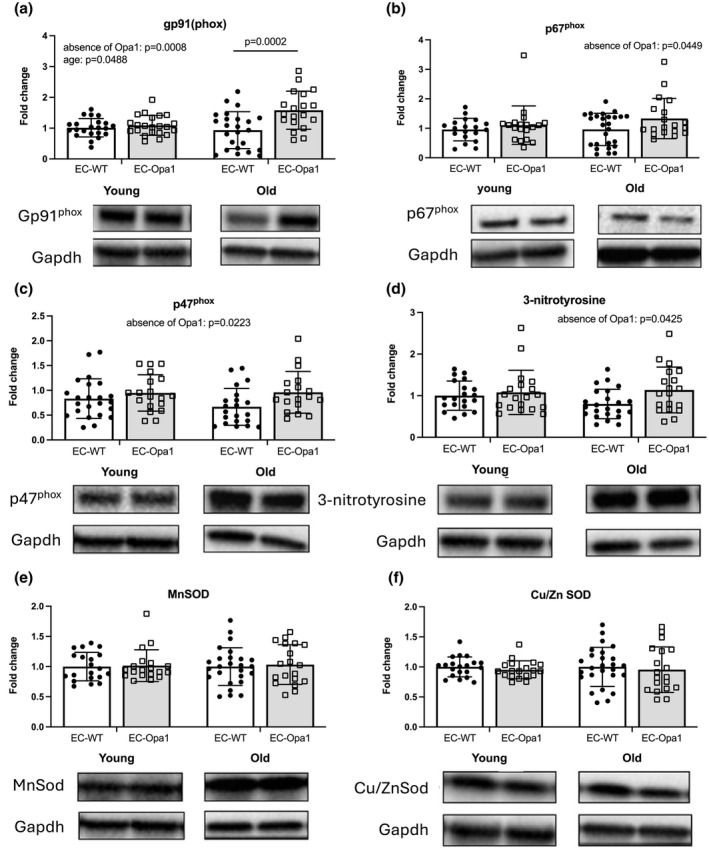
Protein expression levels of Gp91^phox^, p67^phox^, p47^phox^, 3‐nitrotyrosine, Mn superoxide dismutase (Sod) and Cu/ZnSod in the kidney. Protein expression levels of gp91^phox^ (a), p67^phox^ (b), p47^phox^ (c), 3‐nitrotyrosine (d), MnSod (e) and Cu/ZnSod (f) was determined in kidneys isolated from young and old EC‐WT and EC‐Opa1 mice. Mean ± SD is shown (*n* = 21–27 mice per group). Young and old samples came from separate blots. Complete blot panels with marker proteins can be viewed in the Figure S8. Two‐way ANOVA and Bonferroni's multiple comparisons test.

The expression level of Gp91^phox^ in kidneys was significantly increased in old EC‐Opa1 mice compared to old EC‐WT mice (Figure 7a) whereas no significant change in Gp91^phox^ expression level was observed in young EC‐Opa1 mice compared to young EC‐WT mice.

The ANOVA analysis identified a significant effect of the absence of Opa1 in p47^phox^, p67^phox^, and 3‐NT expression levels, without a significant effect of age (Figure 7b–d).

No significant difference between groups was found for Mn superoxide dismutase (MnSod) and Cu superoxide dismutase (Cu/ZnSod) (Figure 7e,f).

### 3.6 Comparison of male and female mice

In the data described above (Figures 1, 2, 3, 4, 5, 6, 7) male and female mice were pooled. In the Figures S1–S7, data from male and female mice are separated. No significant difference was observed between male and female mice.

## 4 DISCUSSION

The present study shows that reduced Opa1 expression in ECs induces hypercontractility and reduces endothelium‐dependent relaxation in resistance arteries in old mice. These observations were associated with hyperuremia and increased expression of markers of oxidative stress in kidneys.

We used 20‐month‐old mice, not older, to avoid the occurrence of important changes in vascular reactivity and kidney function, assuming that changes due to the reduced expression in Opa1 in ECs would be more difficult to observe in older mice with too many organ dysfunctions. Indeed, 20‐month‐old EC‐WT mice had no significant change in vascular reactivity and no obvious alteration in kidney function, as shown by the absence of difference between young and old EC‐WT mice. Under these conditions, changes observed in old EC‐Opa1 mice should come mainly from the reduction in Opa1 expression in ECs.

In a previous study, we have shown that in this model (EC‐Opa1 mice), Opa1 protein expression is reduced by 61% in ECs (Chehaitly et al., 2022). In this study, flow‐mediated dilation was reduced in both mesenteric resistance arteries and in isolated perfused kidneys. This was associated with increased expression of markers of oxidative stress. No difference was observed between male and female mice (Chehaitly et al., 2022). Thus, in the present study, we pooled male and female mice.

Kidney disease is a major public health problem worldwide and its importance is increasing (Francis et al., 2024). The kidney has one of the richest ECs populations linked to a dense microcirculation. The kidney is also a target organ very early affected in CVD (Jourde‐Chiche et al., 2019). Thus, our results showing reduced endothelium‐dependent relaxation and increased markers of oxidative stress in the kidney of 20‐month‐old EC‐Opa1 mice support the assumption that changes in endothelial function affect early kidney function.

Although ECs use mainly glycolysis to produce ATP, they have a major role in ECs function. Endothelial Opa1 has a role in ECs responsiveness to flow (shear stress) as shown by our previous work (Chehaitly et al., 2022) and is necessary for angiogenesis (Herkenne et al., 2020). Furthermore, reduced Opa1 expression enhanced angiotensin II‐induced hypertension in *Opa1* ^+/−^ mice (Robert et al., 2021). In old EC‐Opa1 mice, we found a stronger alteration in vascular reactivity with hypercontractility and reduced endothelium‐dependent relaxation. In young EC‐Opa1 mice, this was not observed, in agreement with our previous work (Chehaitly et al., 2022) showing only a reduction in flow‐mediated dilation in mesenteric arteries isolated from EC‐Opa1 mice. Interestingly, it has been demonstrated that a significant decrease in Opa1 expression in coronary endothelial cells in a type 1 diabetes mouse model possibly contributes to excessive mitochondrial fission and mitochondrial ROS production (Makino et al., 2010).

In the kidney of old EC‐Opa1 mice, we found an increase in Cav‐1 expression level. This observation agrees with a previous work showing that ROS formation increases Cav‐1 expression (Ciurica et al., 2019). Thus, in the present study, Cav‐1 expression increased in old EC‐Opa1 mice kidneys possibly because of excessive ROS formation. Furthermore, Cav‐1 binds to eNos leading to its inactivation, and thus the increased Cav‐1 expression could take part in the reduction in endothelium‐mediated relaxation observed in old EC‐Opa1 mice. In addition, a previous work has shown that an increase in Cav‐1 expression is correlated with acute kidney disease (Cesareo et al., 2022). This is in line with our results showing an increase in blood urea in aged EC‐Opa1 mice. Of note, targeting Cav‐1 is also a promising therapeutic route in chronic kidney disease (Luo et al., 2021).

In EC‐Opa1 aged kidney, we observed a significant increase in the fission protein Fis1 expression level without any change in Mfn2 expression, the other protein involved in the mitochondrial network, suggesting an absence of a compensatory mechanism with a possible increase in mitochondrial fission. Fis1 is involved in mitochondrial fission, which produces smaller mitochondria that will then undergo mitophagy (Kleele et al., 2021). Our findings showing an excessive expression of markers of oxidative stress and vascular disorders in mice lacking ECs Opa1 agree with previous studies showing that a disequilibrium between fusion and fission occurs in cardiovascular diseases (Freed et al., 2014; Kadlec et al., 2016; Marin‐Garcia & Akhmedov, 2016; Ong et al., 2010).

In addition, we observed a significant increase in Pgc‐1α expression in the kidney of old EC‐Opa1 mice. Pgc‐1α is a major regulator of mitochondrial biogenesis, and the reduced OPA1 expression in EC‐Opa1 mice could induce an increase in Pgc‐1α as a compensatory mechanism. Indeed, increasing Pgc‐1α expression in renal tubular cells improves energy production and protects the kidney, whereas increasing Pgc‐1α level in ECs deteriorates endothelial function (Li & Susztak, 2018). The results of the present study do not allow discerning ECs from the other cell types in the kidney, as the whole kidney was used for the western‐blot analysis. Thus, we can only speculate that the compensatory mechanism is not protective enough to prevent oxidative stress in EC‐Opa1 mice. Nevertheless, mitochondrial biogenesis, the formation of new functional mitochondria, is an important defense system for cells to overcome mitochondrial damages (Lenaers et al., 2012) and Pgc‐1α could also be activated by external stimuli such as ROS (Votruba et al., 2003).

Surprisingly, this increase in Pgc‐1α was not followed by an increase in Nrf‐1, but its expression level decreased, suggesting a possible deregulation in the activation chain of the genes that are important for normal mitochondrial functioning. Nevertheless, Nrf‐1 regulates genes involved in the antioxidant defense pathways of the cell. Indeed, Nrf‐1 binds to the antioxidant response element and regulates the expression of enzymes involved in glutathione biosynthesis and other oxidative defense enzymes (Biswas & Chan, 2010; Ohtsuji et al., 2008). Thus, the Nrf‐1 overexpression observed in the present study could reflect a response to excessive oxidative stress in old EC‐Opa1 mice. This issue remains to be further explored.

Furthermore, the absence of endothelial Opa1 in old EC‐Opa1 mice kidneys was associated with an increased expression of membrane components of NADPH oxidases Gp91, suggesting higher ROS formation. Two other NADPH oxidase subunits, p47 and p67, were increased in EC‐Opa1 mice independently of age. Thus, the absence of endothelial Opa1 possibly induces oxidative stress, which could likely be exacerbated by aging. This was not followed by an increase in Sod expression, as we did not disclose a change in MnSod and Cu/ZnSod expression. These findings suggest that the absence of Opa1 in aged kidneys could lead to oxidative stress without a compensatory protective action by Sod. This increased expression in NADPH oxidase subunits was associated with increased 3‐nitrotyrosine levels in the kidneys of EC‐Opa1 mice, thus confirming that oxidative stress could occur in the absence of Opa1. Increased 3‐nitrotyrosine has been previously observed in aged and diabetic mice (Sakul et al., 2013). Indeed, protein tyrosine nitration is a good marker of oxidative stress leading to alteration of the activity of the nitrated proteins (Xu et al., 2006). Our observations agree with our previous work showing increased oxidative stress and inflammation in the kidneys of old mice (Guivarc'h et al.) and with another study showing that silencing Fis1 or Drp1 reduced high glucose‐induced alterations in mitochondrial ROS production, indicating that increasing mitochondrial fission could be negative for endothelial function due to an increase in ROS production (Cesareo et al., 2022).

Nevertheless, a limitation of the present study is that we did not measure directly oxidative stress in blood vessels and in the kidneys. Another limitation is that vascular reactivity was determined in mesenteric arteries and not directly measured in renal arteries. Thus, further studies are needed to strengthen our hypothesis. It should also be noted that comparisons for the same protein between young and old samples were made on separate blots and that this is a weakness of the present study.

In conclusion, we demonstrate that endothelial Opa1 has a protective effect during aging by maintaining vascular and kidney function possibly through the reduction of oxidative stress.

These findings suggest that improving mitochondrial fusion or, more generally, mitochondrial dynamics could propose a new target for a therapeutic approach against endothelial disorders related to aging and might protect the vascular tree in target organs such as the kidney.

## AUTHOR CONTRIBUTIONS

Daniel Henrion conceived and designed research. Carlotta Turnaturi, Loïck L'Hoste, Coralyne Proux, Linda Grimaud, Emilie Vessieres, and Anne Teissier performed experiments. Daniel Henrion, Carlotta Turnaturi, Loïck L'Hoste, Coralyne Proux, Linda Grimaud, Emilie Vessieres analyzed data. Daniel Henrion, Carlotta Turnaturi, Loïck L'Hoste, Coralyne Proux, Linda Grimaud, Emilie Vessieres interpreted results of experiments. Daniel Henrion, Carlotta Turnaturi, Coralyne Proux, Linda Grimaud, Emilie Vessieres, and Loïck L'Hoste prepared figures. Daniel Henrion, Carlotta Turnaturi, Coralyne Proux, Linda Grimaud, Emilie Vessieres, and Loïck L'Hoste drafted manuscript. Daniel Henrion, Carlotta Turnaturi, Antonio Zorzano, Pascal Reynier, Raffaella Sorrentino, Guy Lenaers, Laurent Loufrani, and Loïck L'Hoste edited and revised manuscript. Daniel Henrion, Carlotta Turnaturi, Loïck L'Hoste, Coralyne Proux, Linda Grimaud, Emilie Vessieres, Anne Teissier, Antonio Zorzano, Pascal Reynier, Raffaella Sorrentino, Guy Lenaers, and Laurent Loufrani approved final version of manuscript.

## FUNDING INFORMATION

This study was supported by the University of Angers (Angers, France), the INSERM (Institut National pour la Santé et la Recherche Médicale, Paris, France) and the CNRS (Centre National de la Recherche Scientifique, Paris, France).

## ETHICS STATEMENT

All mouse studies were approved by the local Institutional Animal Care and Use Committe of “Pays de la Loire”.

## Supporting information


Data S1.

## Data Availability

The data presented in this study are available on request from the corresponding author.
