# Supplementary material for: Altered endothelial mitochondrial Opa1‐related fusion in mouse accelerates age‐associated vascular and kidney damage
Source: Physiol Rep. 2025 Jul 4;13(13):e70451. doi: 10.14814/phy2.70451 (PMC12227657; doi:10.14814/phy2.70451)
Supplement: Supplementary file 1 — Data S1. [file PHY2-13-e70451-s001.pdf]

Altered endothelial mitochondrial Opa1-related fusion in mouse accelerates age-associated kidney damage

Carlotta Turnaturi, Loïck L'Hoste, Coralyne Proux, Linda Grimaud, Emilie Vessieres, Antonio Zorzano, Anne Teissier, Pascal Reynier, Raffaella Sorrentino, Guy Lenaers, Laurent Loufrani, Daniel Henrion

Supplementary files:

Supplementary table S1: Antibodies used for the Western-blots

Supplementary figures 1 to 7 with male and female mice separated

Supplementary Figure S8: Uncropped Western blots

Supplementary table S1: Antibodies used for the Western-blots

Target antigen	Vendor or Source	Catalog #	Working concentration	URL
eNOos	BD bioscience	BD610297	1/1000	https://www.bdbiosciences.com/en-us/products/reagents/microscopy-imaging-reagents/immunofluorescence-reagents/purified-mouse-anti-enos-nos-type-iii.610297?tab=product_details
p47 phox	BD bioscience	BD610355	1/500	https://www.bdbiosciences.com/zh-cn/products/reagents/microscopy-imaging-reagents/immunofluorescence-reagents/purified-mouse-anti-human-p47-phox.610355?tab=product_details
P67 phox	Abclonal	A1178	1/1000	https://eutemp.abclonal.com/catalog-antibodies/NOXA2p67phoxRabbitpAb/A1178
gp91 phox	BD bioscience	BD611414	1/1000	https://www.bdbiosciences.com/en-us/products/reagents/microscopy-imaging-reagents/immunofluorescence-reagents/purified-mouse-anti-gp91-phox.611414?tab=product_details
MnSod	Cell signaling	#13141	1/1000	https://www.cellsignal.com/products/primary-antibodies/sod2-d3x8f-xp-rabbit-mab/13141?srsltid=AfmBOoq6Cqzg5z8uLAQ-FhgFeZQzcH8w5DQC59Tn2x_HUh5M7GsOaxsD
Cu/ZnSod	Enzo	ADI-SOS-101-E	1/1000	https://www.enzo.com/product/cu-zn-sod-polyclonal-antibody-2/
Gapdh	Cell signaling	#5174	1/2000	https://www.cellsignal.com/products/primary-antibodies/gapdh-d16h11-xp-rabbit-mab/5174?srsltid=AfmBOopln20qwdaXUYWTYI2LEghfNG9DxMYEVjz1ELSYnwBN5ZObLL6j
Pgc-1α	Abcam	Ab106814	1/1000	https://www.citeab.com/antibodies/745981-ab106814-anti-pgc1-alpha-antibody
Nrf1	Cell signaling	#46743	1/1000	https://www.cellsignal.com/products/primary-antibodies/nrf1-d9k6p-rabbit-mab/46743
Fis-1	Santa Cruz	Sc-376447	1/500	https://www.scbt.com/it/p/fis1-antibody-b-5?srsltid=AfmBOorvM57r7pRoo-jSNh25FpGwFKEGaPRcaJ9fPQXX4eXxIHu4nBxv
Mfn2	Novus biologicals	H00009927-M01	1/1000	https://www.novusbio.com/products/mitofusin-2-antibody-6a8_h00009927-m01?srsltid=AfmBOopsvLdPsg4WZp199Kyr87q3_wda4ygUP0LZCcS0Serivq_IIMw2
3-nitrotyrosine	Abcam	Ab61392	1/1000	https://www.abcam.com/en-us/products/primary-antibodies/3-nitrotyrosine-antibody-39b6-ab61392?srsltid=AfmBOoqlS_3UE6RgXwzsQZZkrQB8EUr43uiGXk_N8FCpvVCNwt_KZe5k
Sirt1	Cell signaling	#9475	1/500	https://www.cellsignal.com/products/primary-antibodies/sirt1-d1d7-rabbit-mab/9475?srsltid=AfmBOorZAqPfKYAo-WwcfasTHWj1e_UfiE-yXXoNKiiwG7ubNLYblYF5
Cav-1	BD bioscience	BD610407	1/1000	https://www.bdbiosciences.com/en-us/products/reagents/microscopy-imaging-reagents/immunofluorescence-reagents/purified-mouse-anti-caveolin-1.610407?tab=product_details
Goat anti-Mouse	TNB72-8062-Moo1	Ozyme	1/4000	https://yris.ozyme.fr/fr/company/ozyme/product/goat-anti-mouse-igg-h-l-affinity-pure-hrp-conjugate-cross-adsorbed-tnb72-8062-1
Goat anti-Rabbit	TNB72-8073-Moo1	Ozyme	1/1000	https://yris.ozyme.fr/fr/company/ozyme/product/goat-anti-rabbit-igg-h-l-f-ab-2-fragment-affinity-pure-hrp-conjugate-cross-adsorbed-tnb72-8074-1

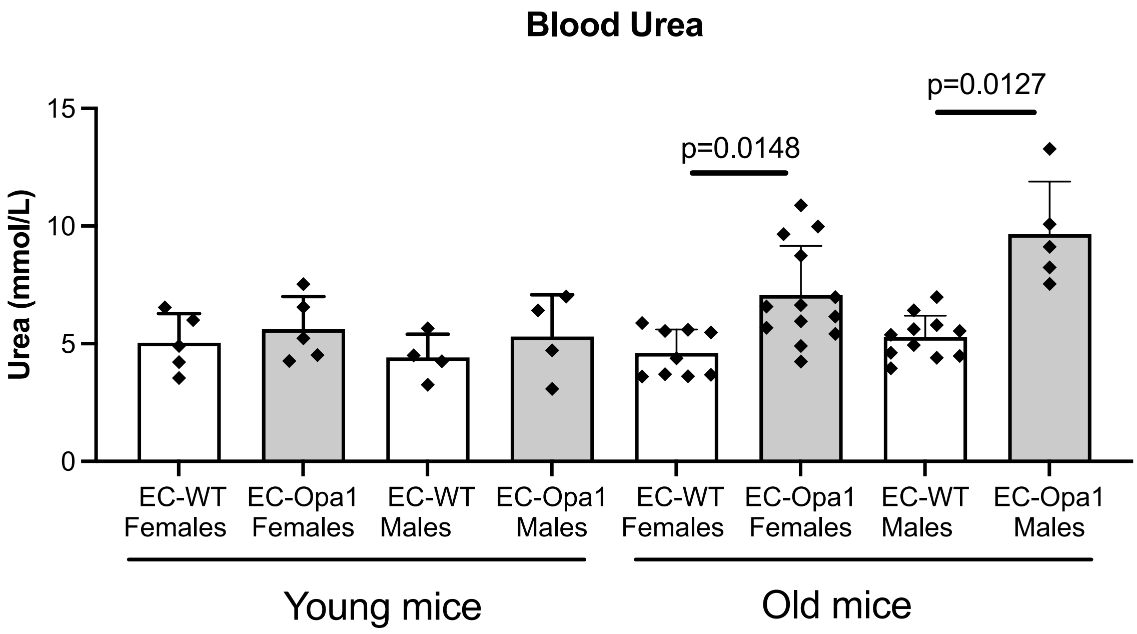

Supplementary figure S1: Blood urea nitrogen measurement

Blood urea nitrogen was measured in young and old male and female EC-Opa1 and EC-WT mice. Data is expressed as means ± SD

Two-way ANOVA and Bonferroni's multiple comparisons test.

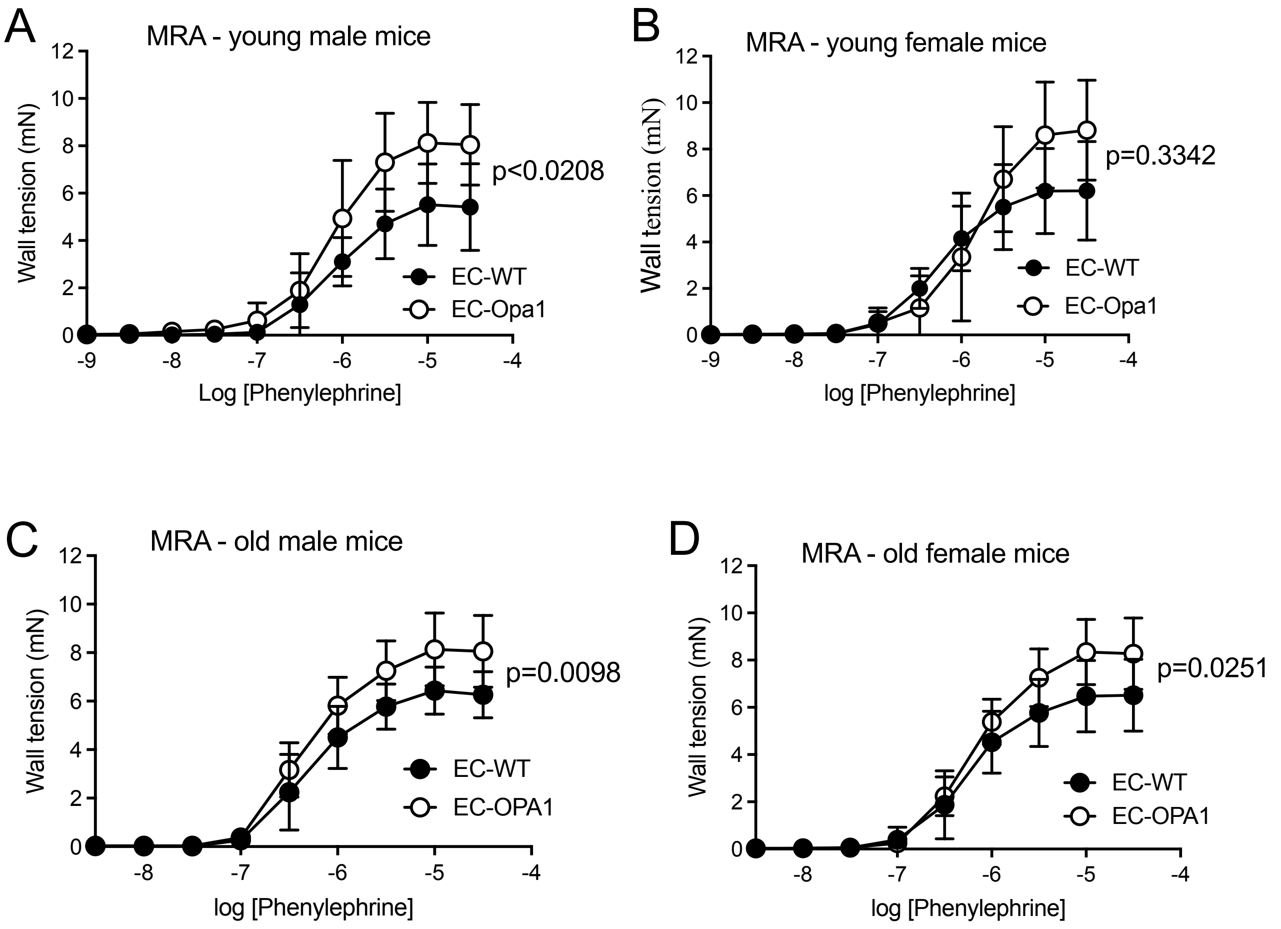

Supplementary figure S2: Phenylephrine-mediated contraction.

Phenylephrine (1 nmol/L to 30 µmol/L)-mediated cumulative concentration-response curve was determined in mesenteric arteries isolated from young (A,B) and old (C,D), male (A,C) and female (B,D), EC-Opa1 and EC-WT mice. Data is expressed as mean ± SD.

Two-way ANOVA for repeated measurements and Bonferroni's multiple comparisons test.

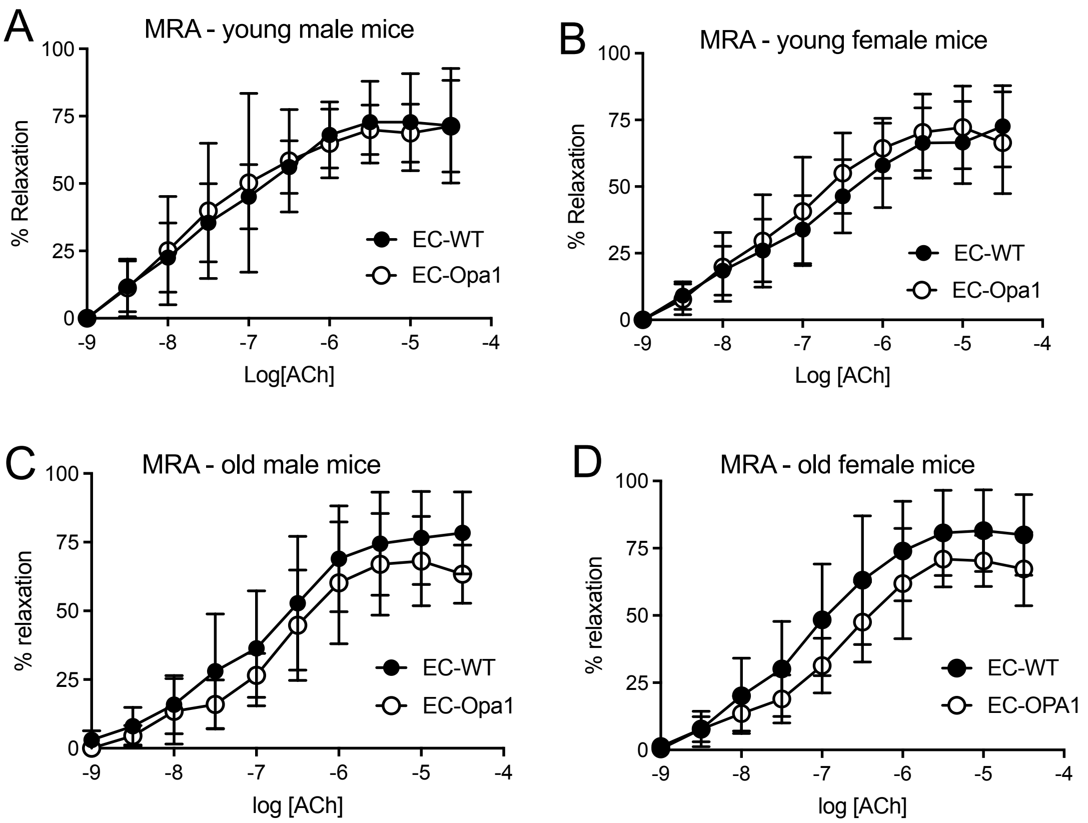

Supplementary figure S3: Acetylcholine-mediated endothelium-dependent relaxation

Acetylcholine (Ach, 1 nmol/L to 30 µmol/L)-mediated endothelium-dependent relaxation was determined in mesenteric arteries isolated from young (A,B) and old (C,D), male (A,C) and female (B,D), EC-Opa1 and EC-WT mice. Data is expressed as means ± SD.

NS, two-way ANOVA for repeated measurements and Bonferroni's multiple comparisons test.

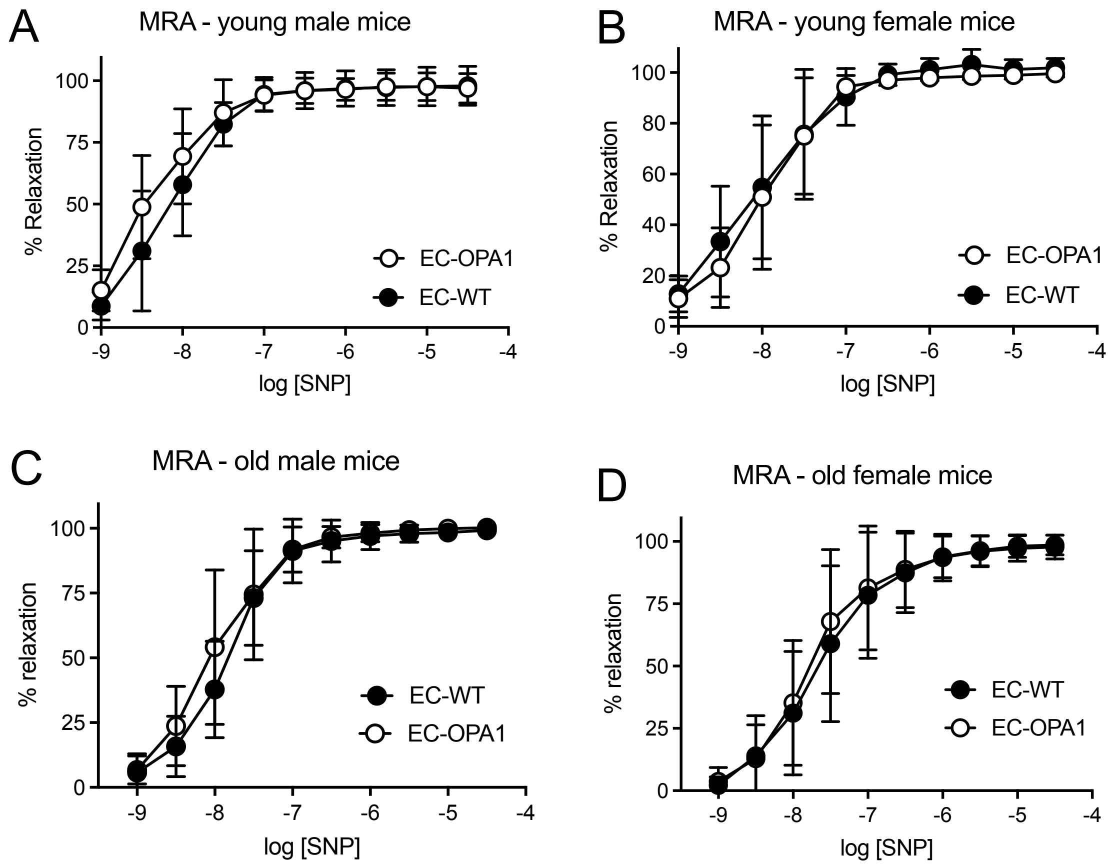

Supplementary figure S4: Sodium nitroprusside-mediated endothelium-independent relaxation

Sodium nitroprusside (SNP, 1 nmol/L to 30 µmol/L)-mediated endothelium-dependent relaxation was determined in mesenteric arteries isolated from young (A,B) and old (C,D), male (A,C) and female (B,D), EC-Opa1 and EC-WT mice. Data is expressed as means ± SD.

NS, Two-way ANOVA for repeated measurements and Bonferroni's multiple comparisons test.

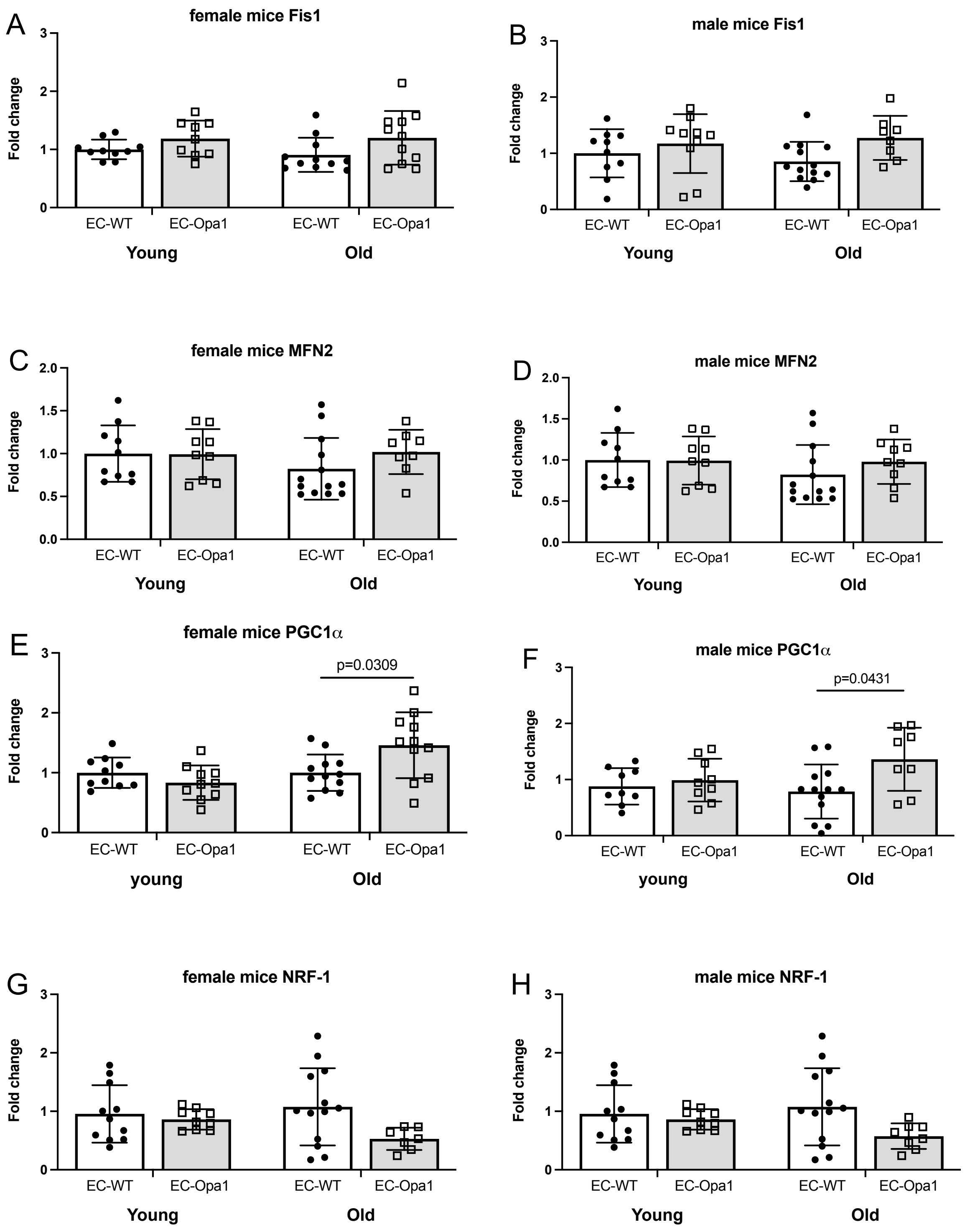

Supplementary figure S5: Protein expression level of Fis1, Pgc1α, Mfn2 and Nrf-2 in the kidney
Protein expression level of Fis1 (A,B), Mfn2 (C,D), Pgc1α (E,F) and Nrf-1 (G,H) was determined in kidneys isolated from young and old male (right panels) and female (left panels) EC-WT and EC-Opa1. Mean±SD is shown.
Uncropped blots are shown in the Supplementary Figure S8.
Two-way ANOVA and Bonferroni's multiple comparisons test.

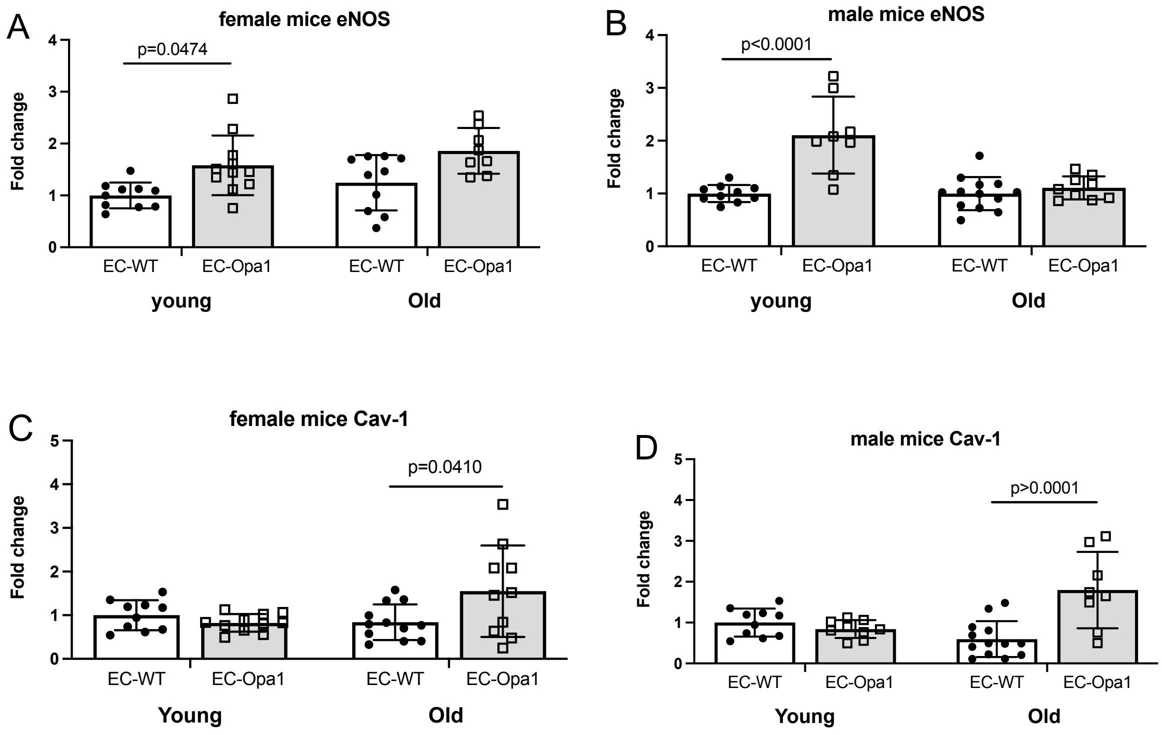

Supplementary figure S6: Protein expression level of eNos and Cav-1 in the kidney
Protein expression level of eNos (A,B) and Cav-1 (C,D) was determined in kidneys isolated from young and old, male (A,C) and female (B,D), EC-WT and EC-Opa1 mice. Mean±SD is shown.
Uncropped blots are shown in the Supplementary Figure S8.
Two-way ANOVA and Bonferroni’s multiple comparisons test.

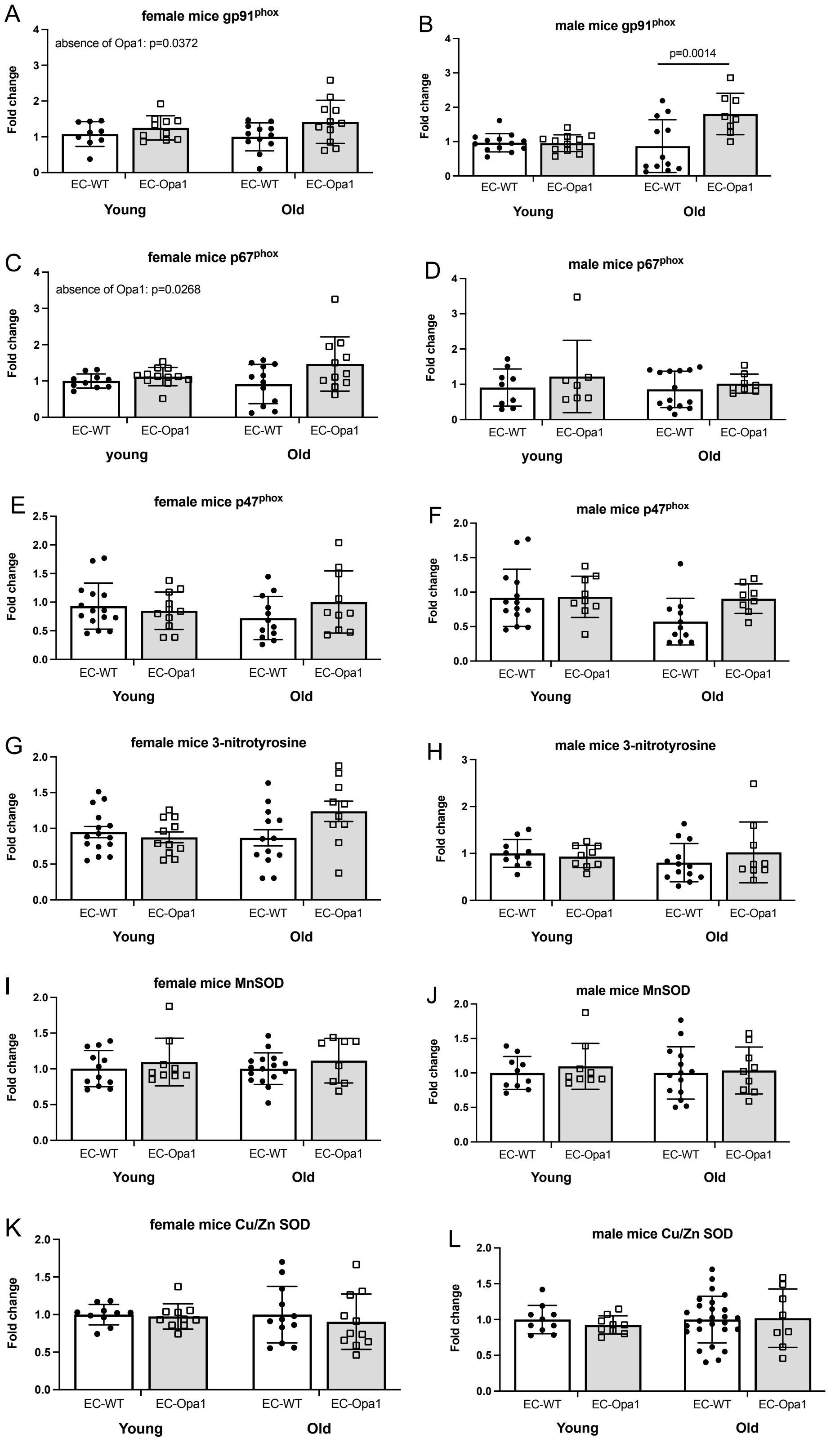

Supplementary figure S7: Protein expression levels of Gp91^{phox} (A,B), p67 ^{phox} (C,D), p47 ^{phox} (E,F), 3-nitrotyrosine (G,H), MnSod (I,J) and Cu/ZnSod (K,L) was determined in kidneys isolated from young and old, male (right panels) and female (left panels), EC-WT and EC-Opa1 mice. Mean±SD is shown.
Uncropped blots are shown in the Supplementary Figure S8.
Two-way ANOVA and and Bonferroni's multiple comparisons test.

Supplementary Figure S8: complete blot panels with marker proteins, corresponding to the data shown in figures 5, 6 and 7 and in the supplementary figure S5 to S7

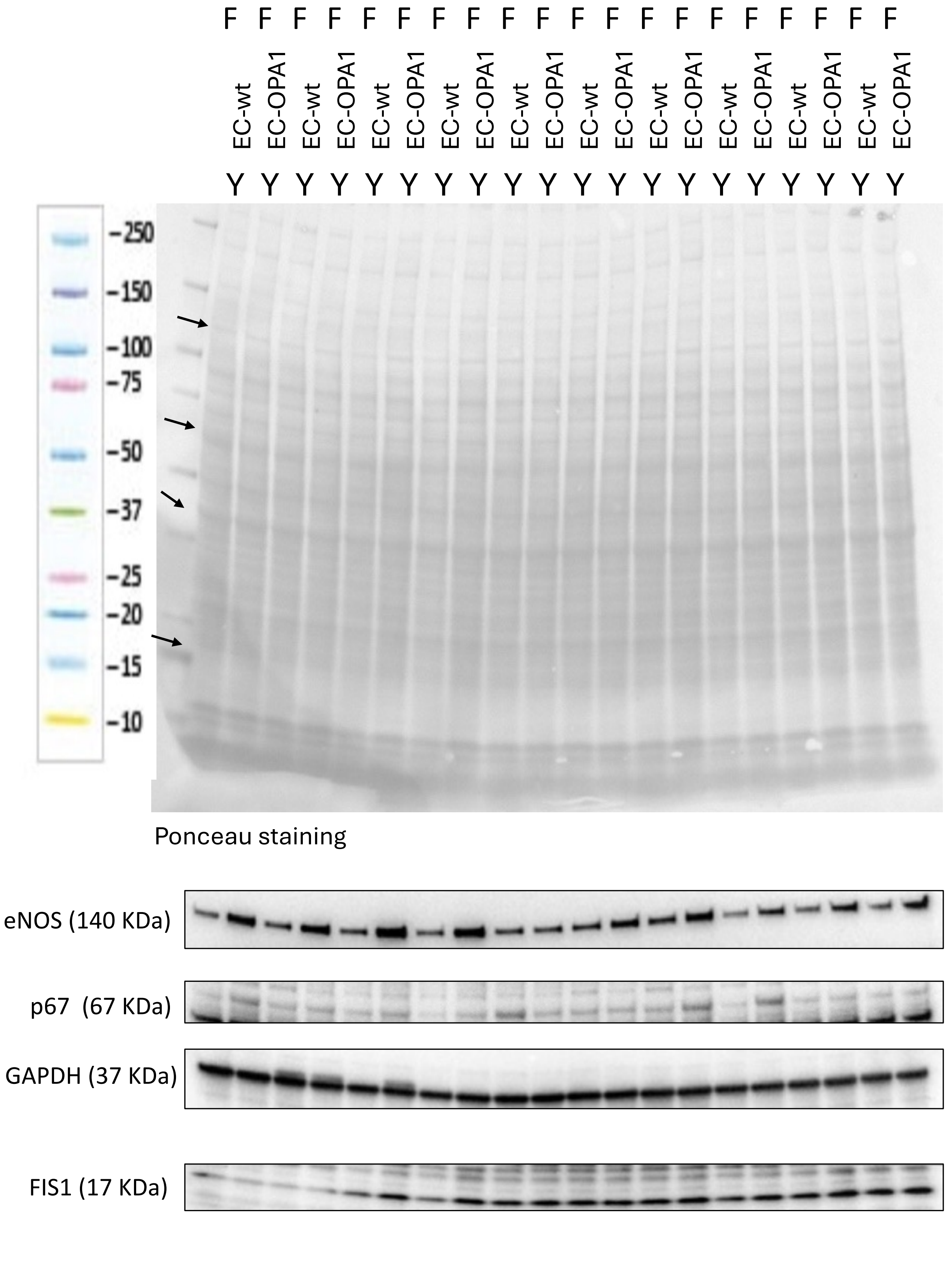

Legend: F = female, Y = young

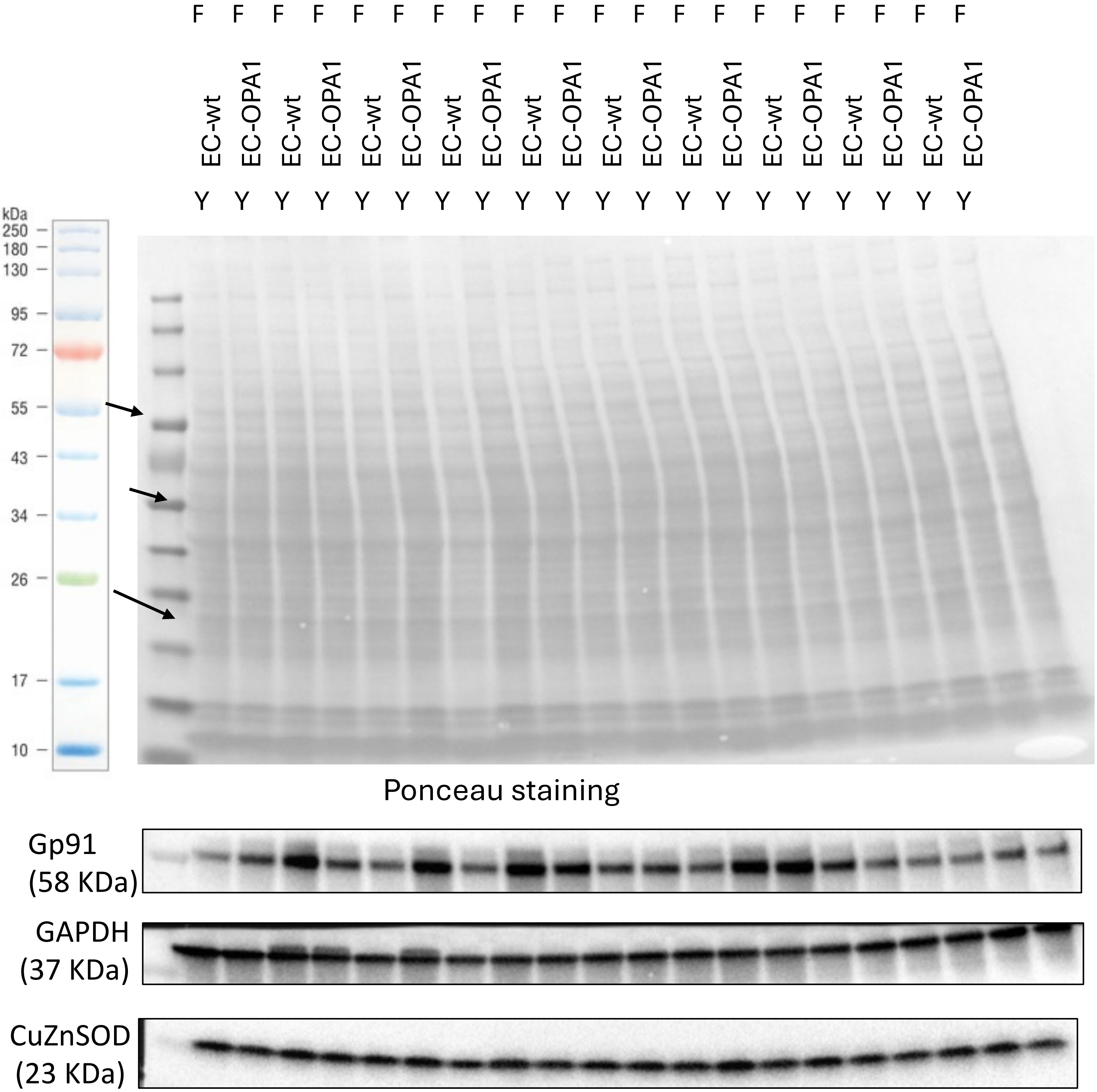

Legend: F = female, Y = young

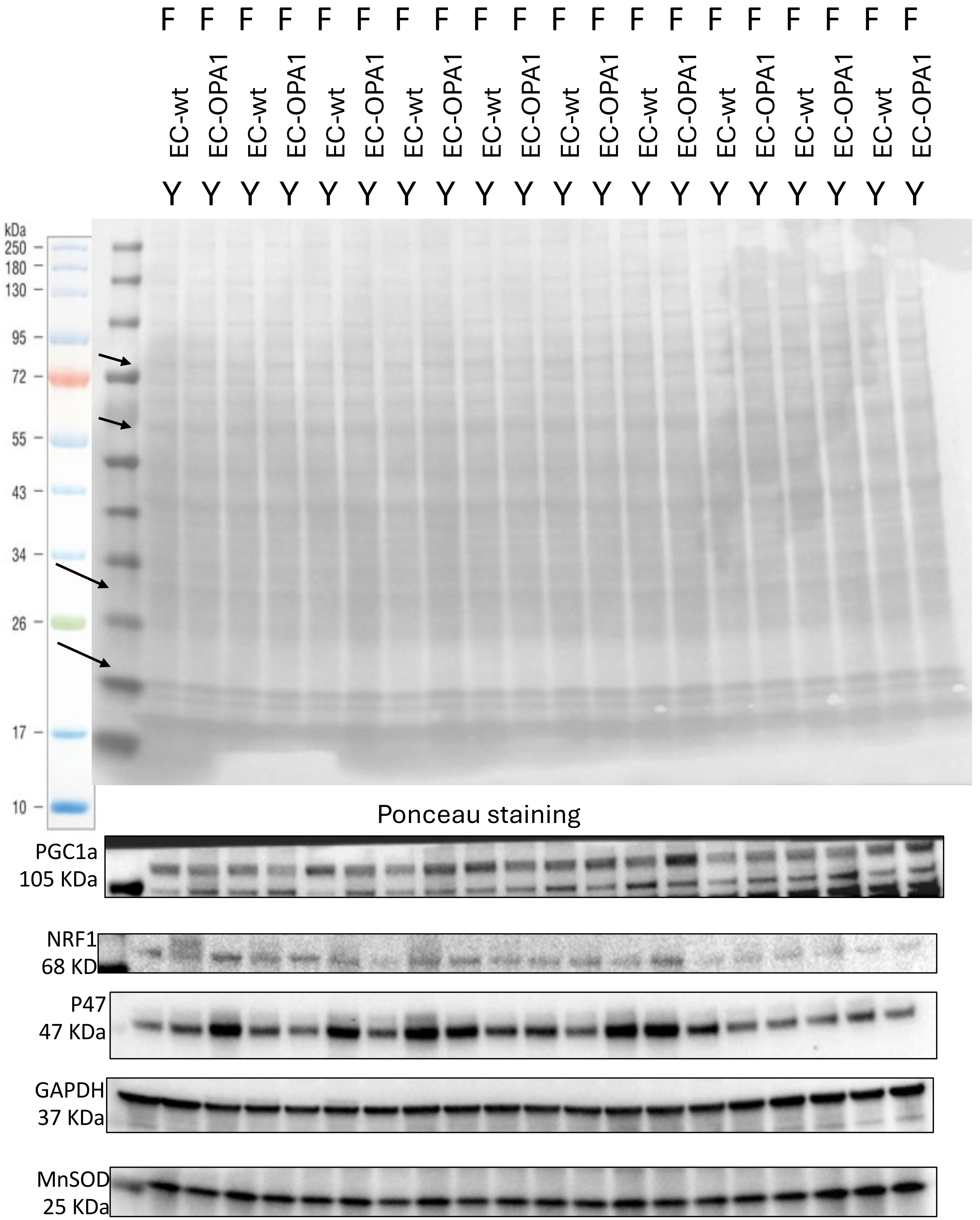

Legend: F = female, Y = young

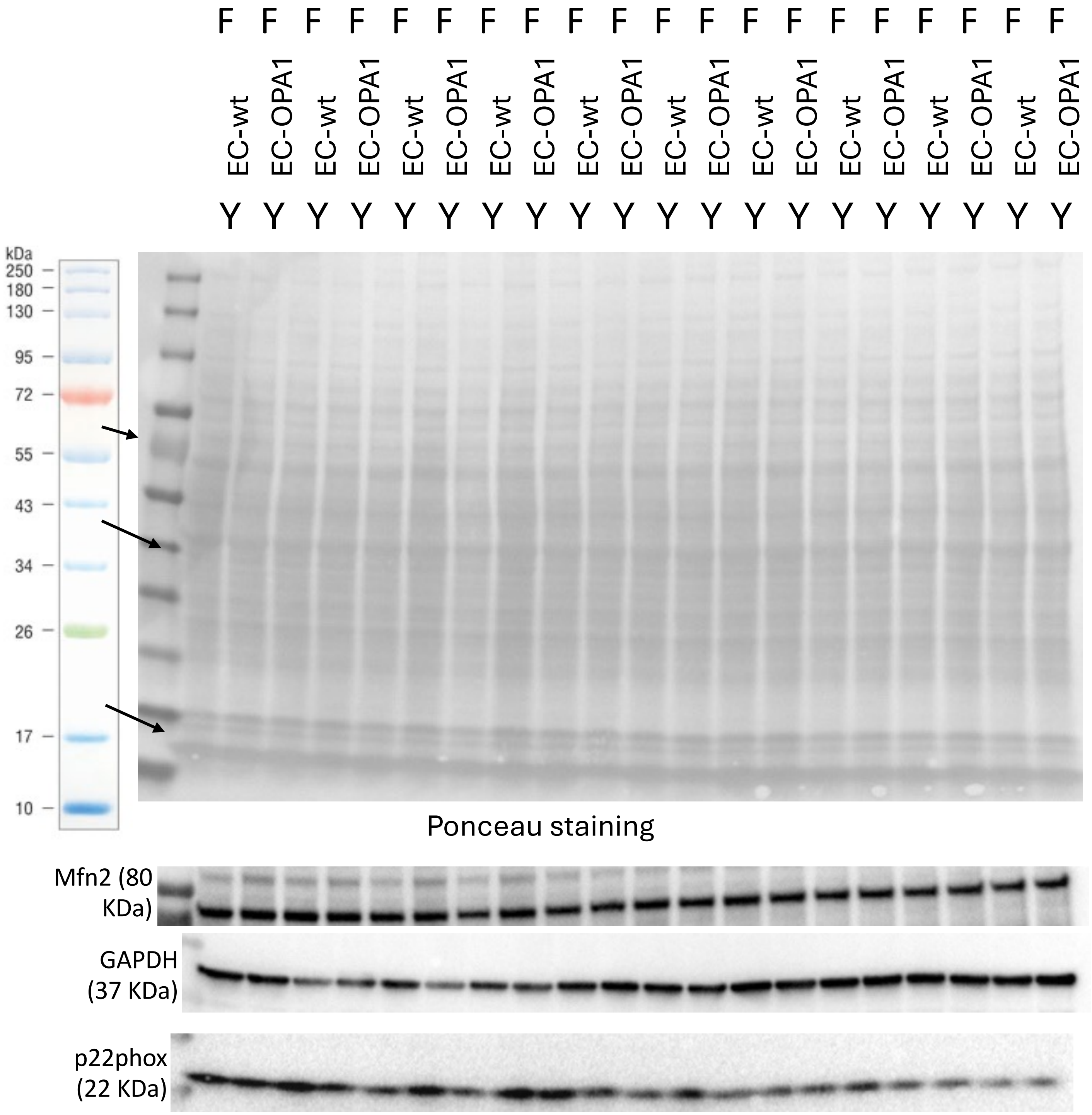

Legend: F = female, Y = young

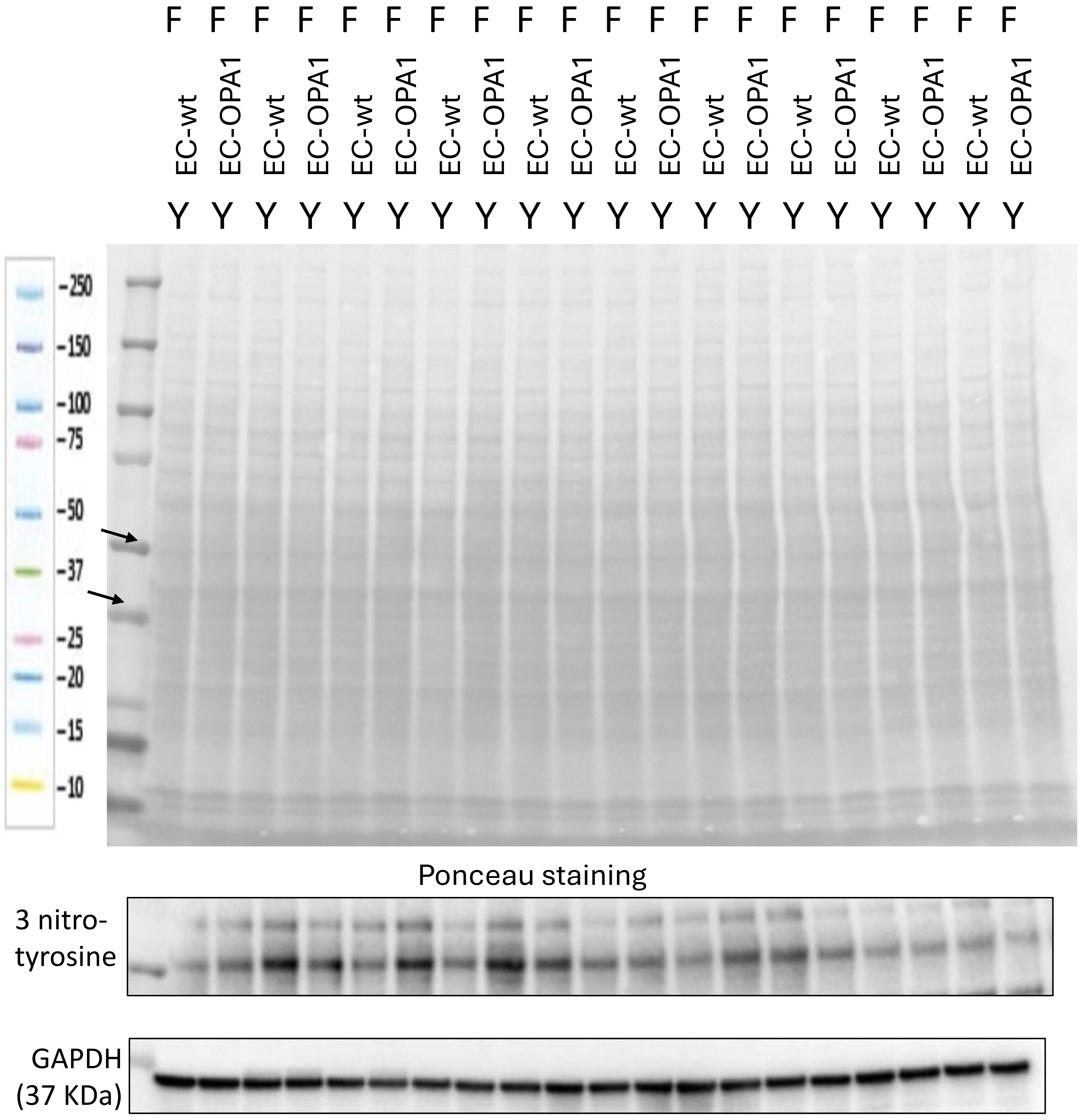

Legend: F = female, Y = young

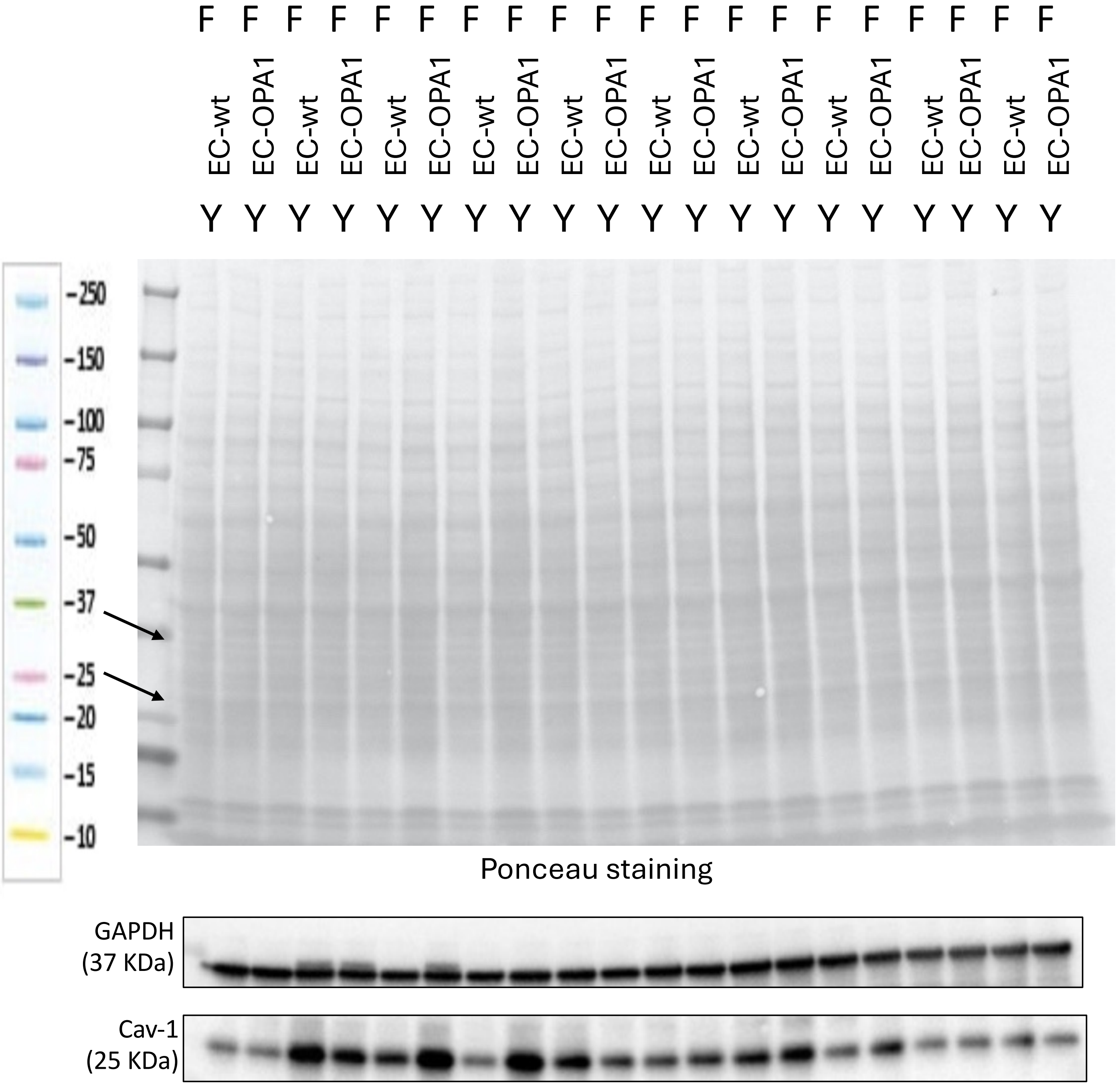

Legend: F = female, Y = young

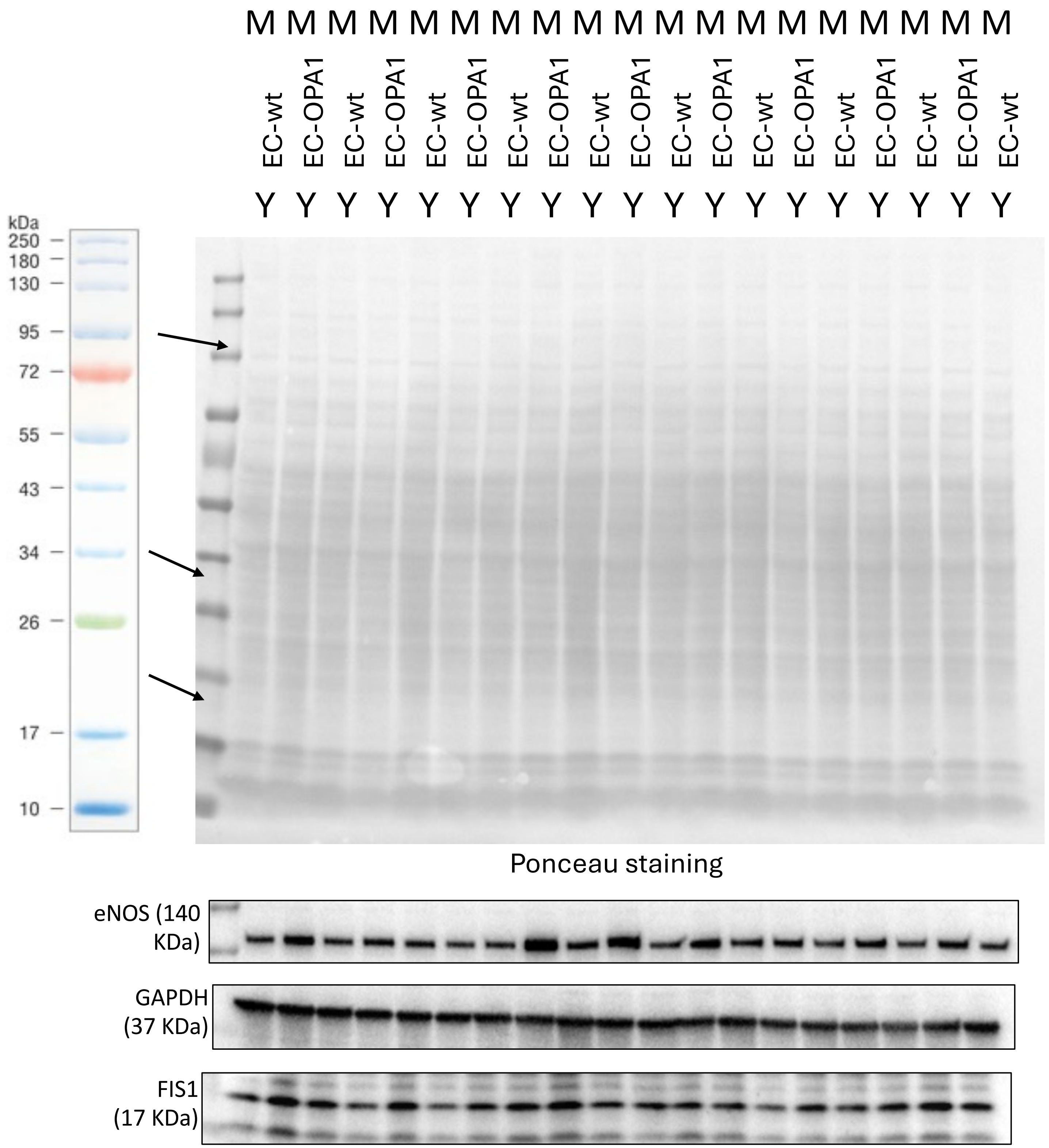

Legend: M = male, Y = young

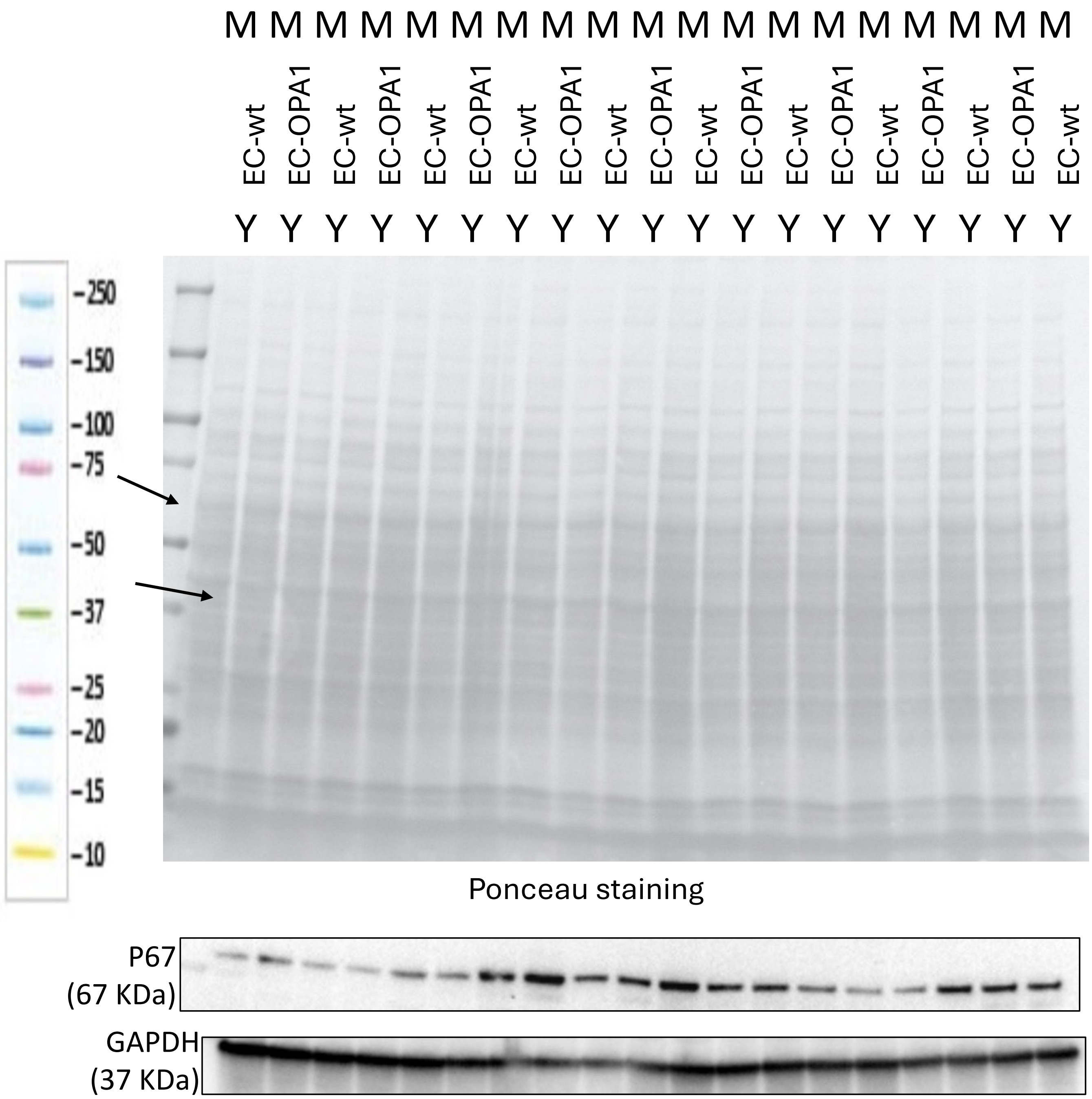

Legend: M = male, Y = young

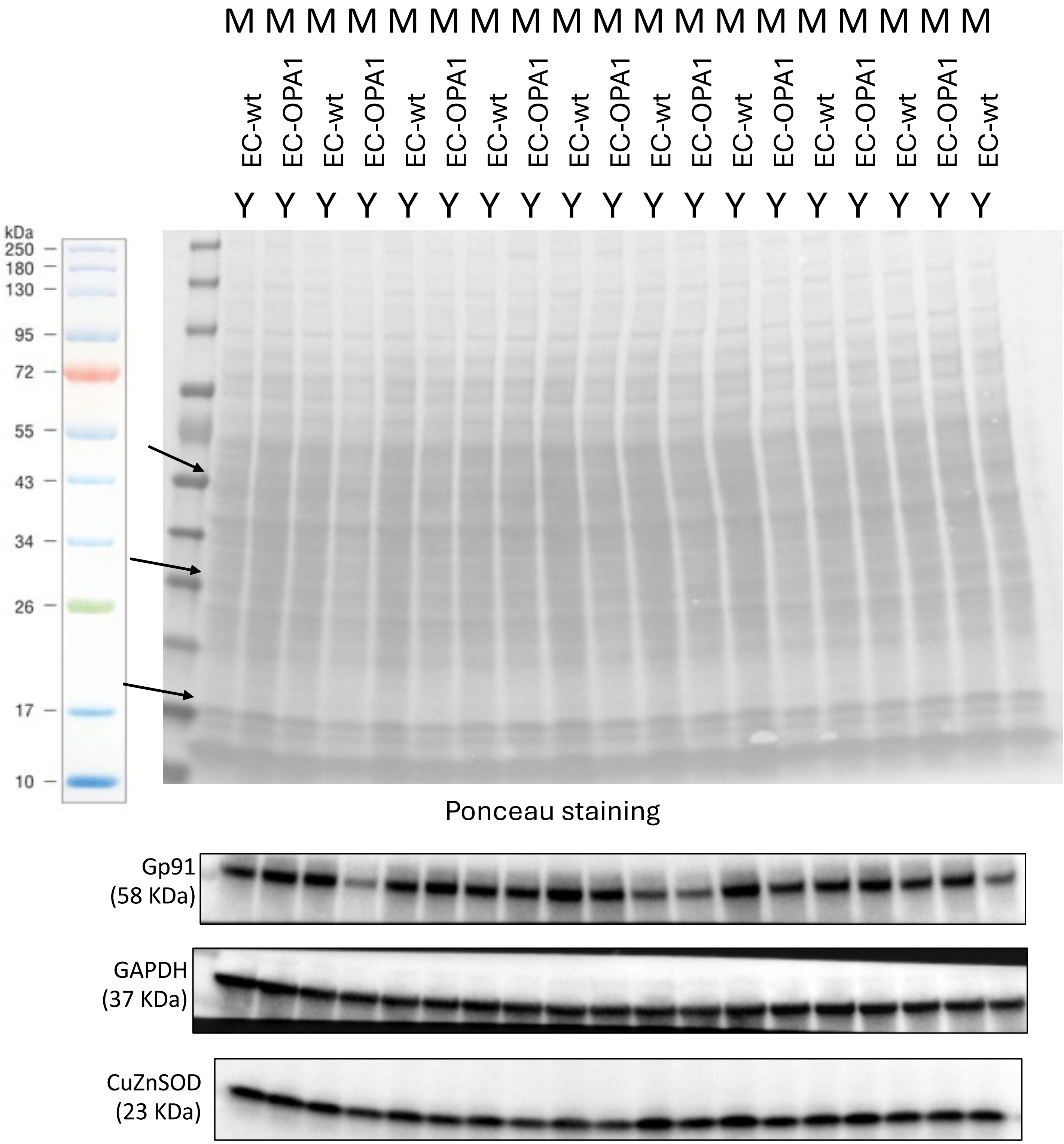

Legend: M = male, Y = young

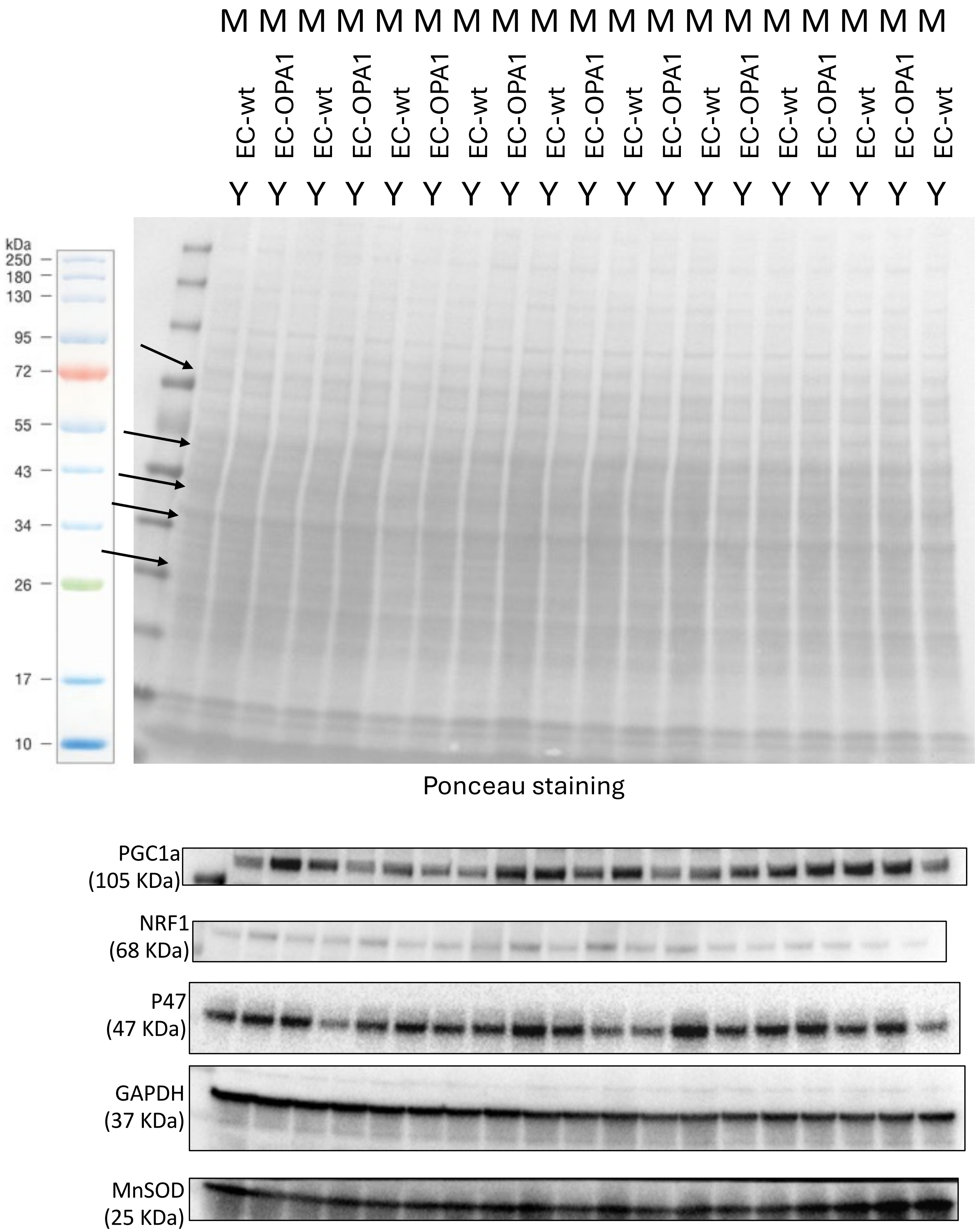

Legend: M = male, Y = young

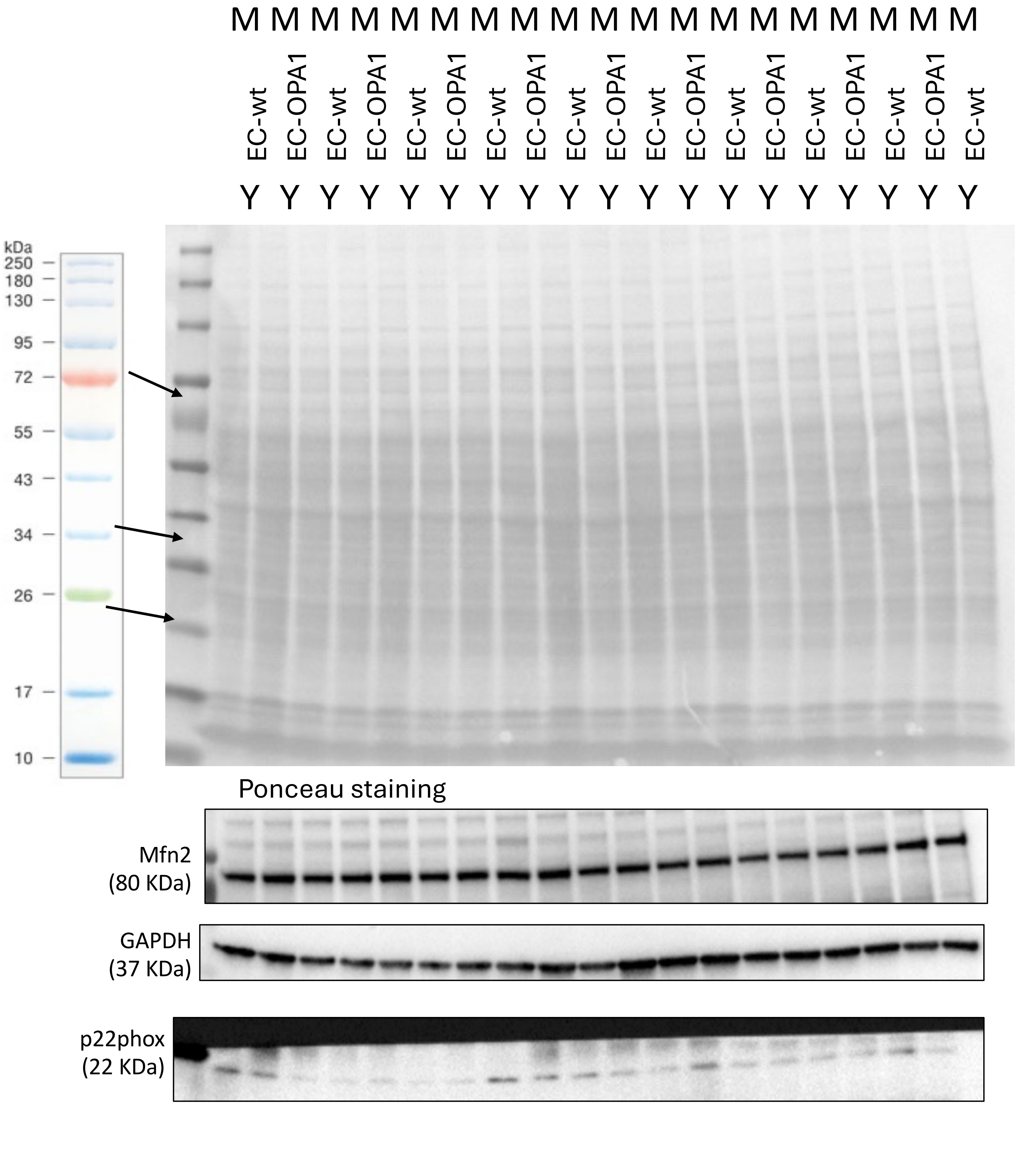

Legend: M = male, Y = young

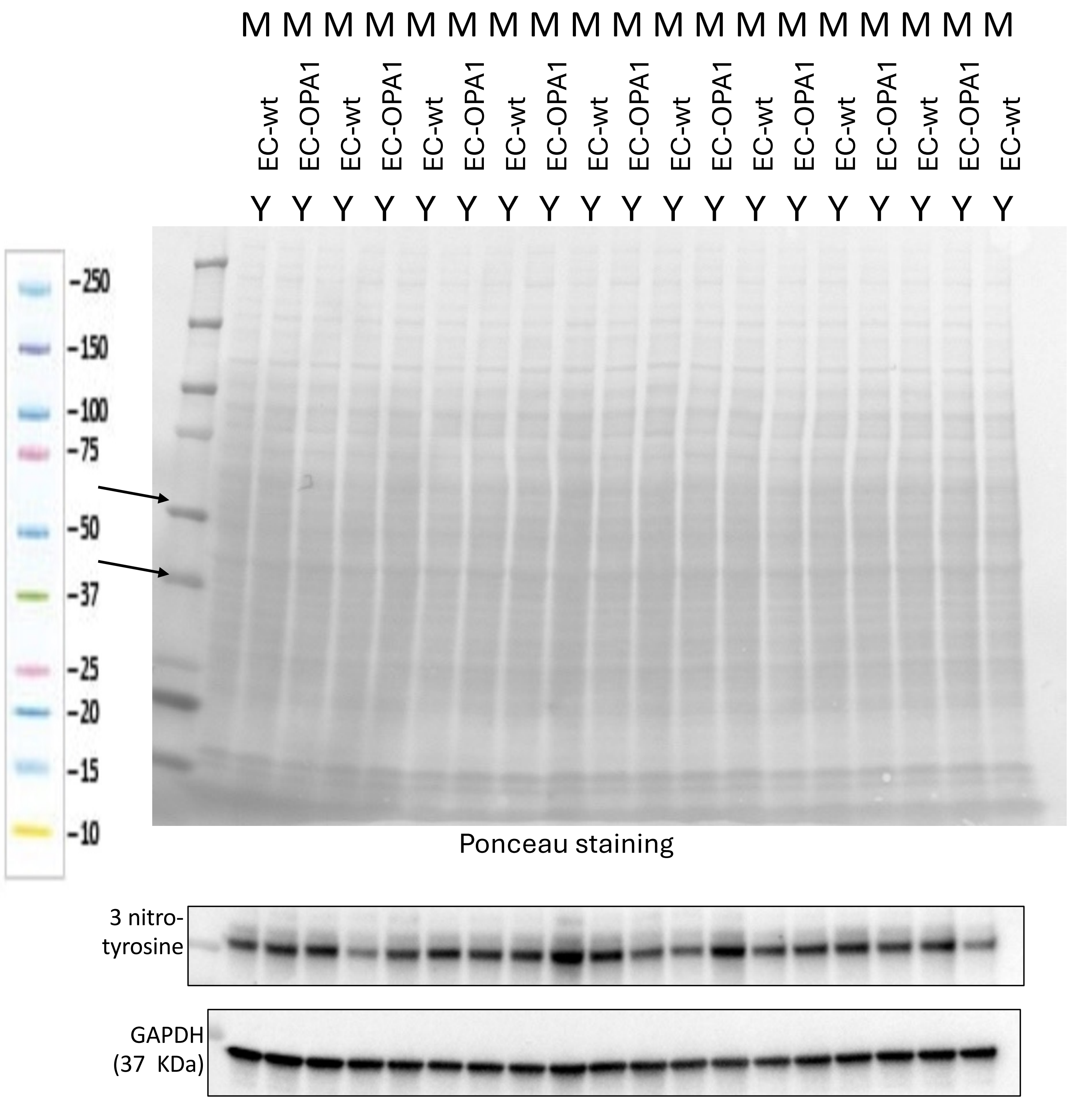

Legend: M = male, Y = young

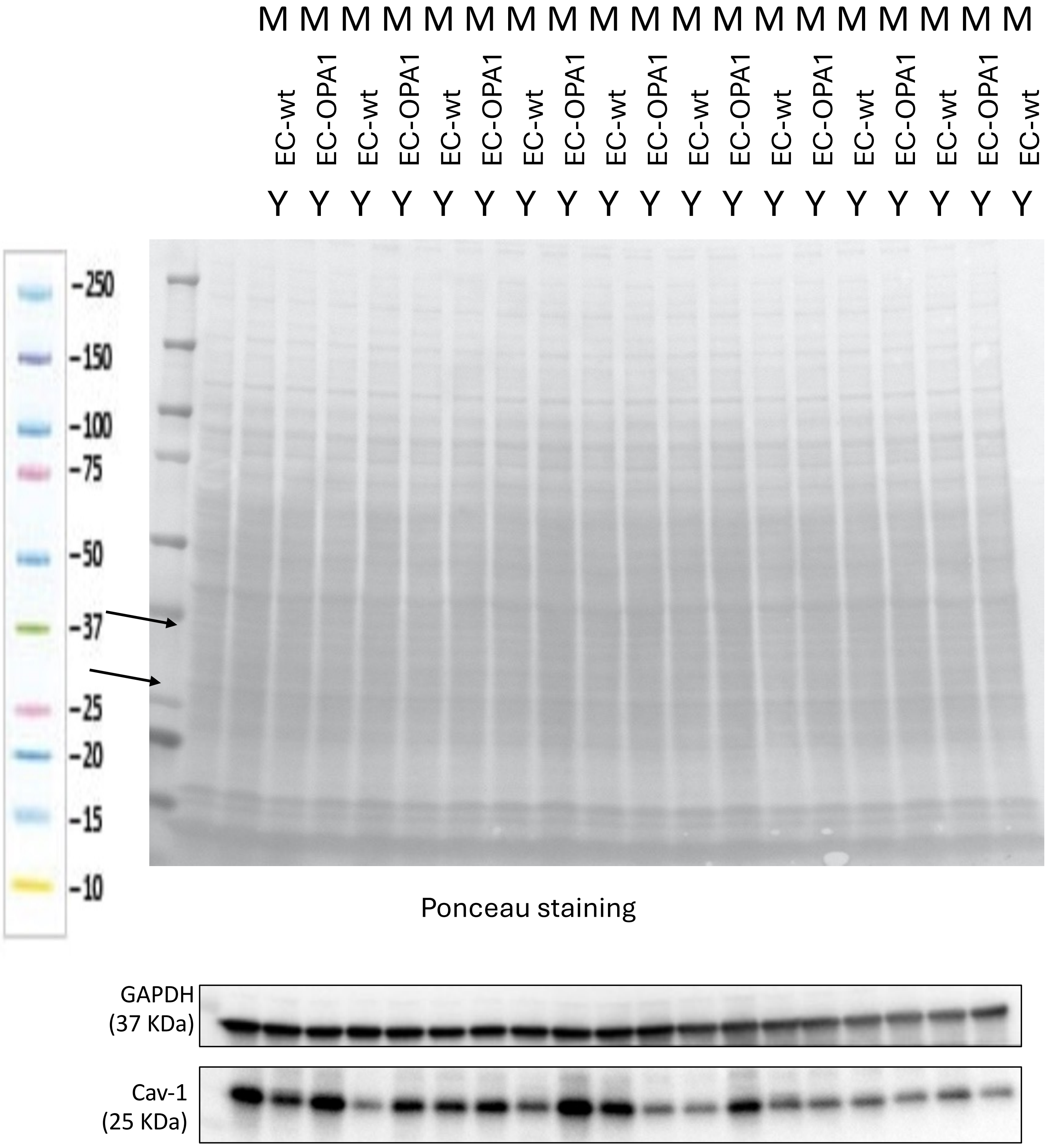

Legend: M = male, Y = young

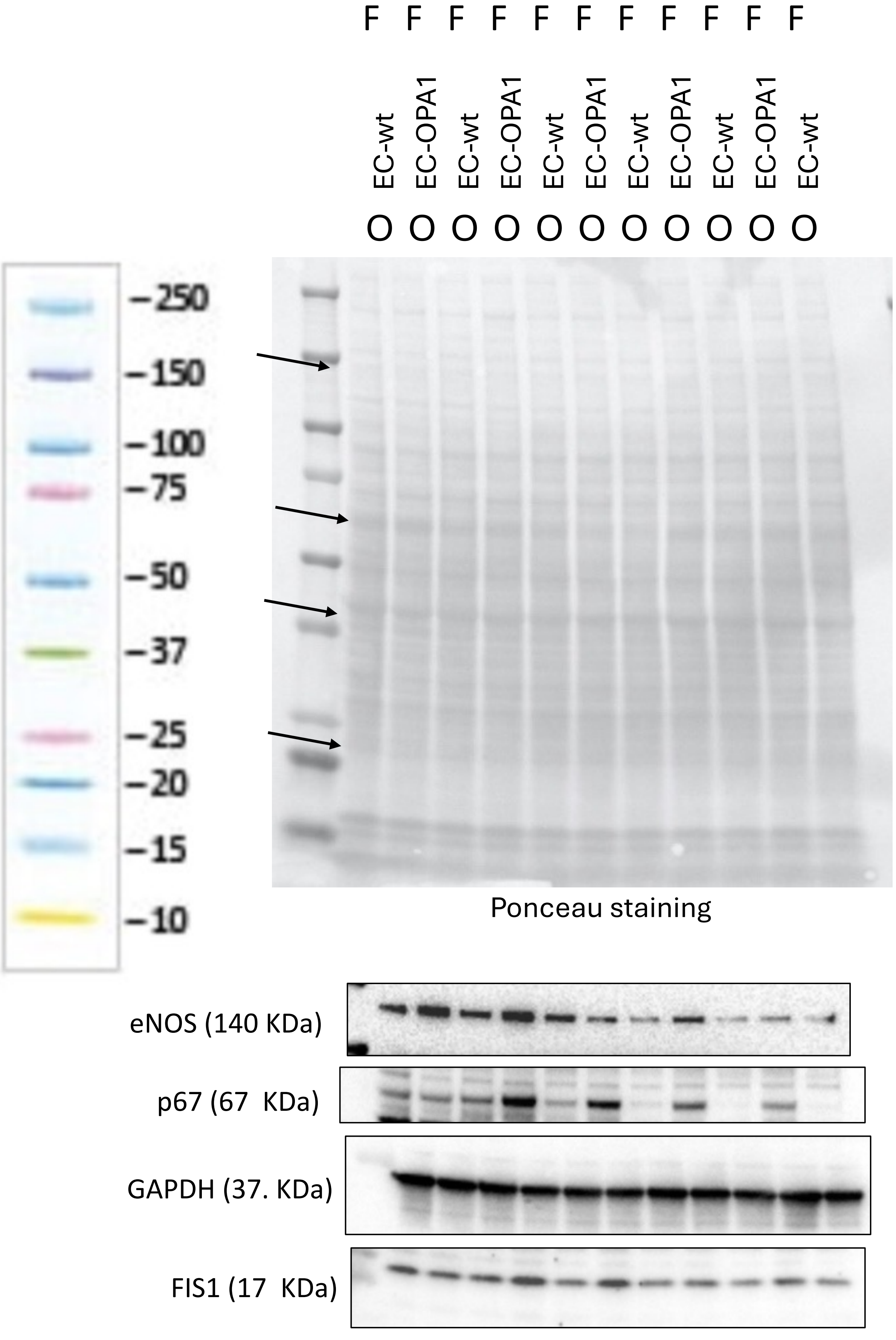

Legend: F = female, O = old

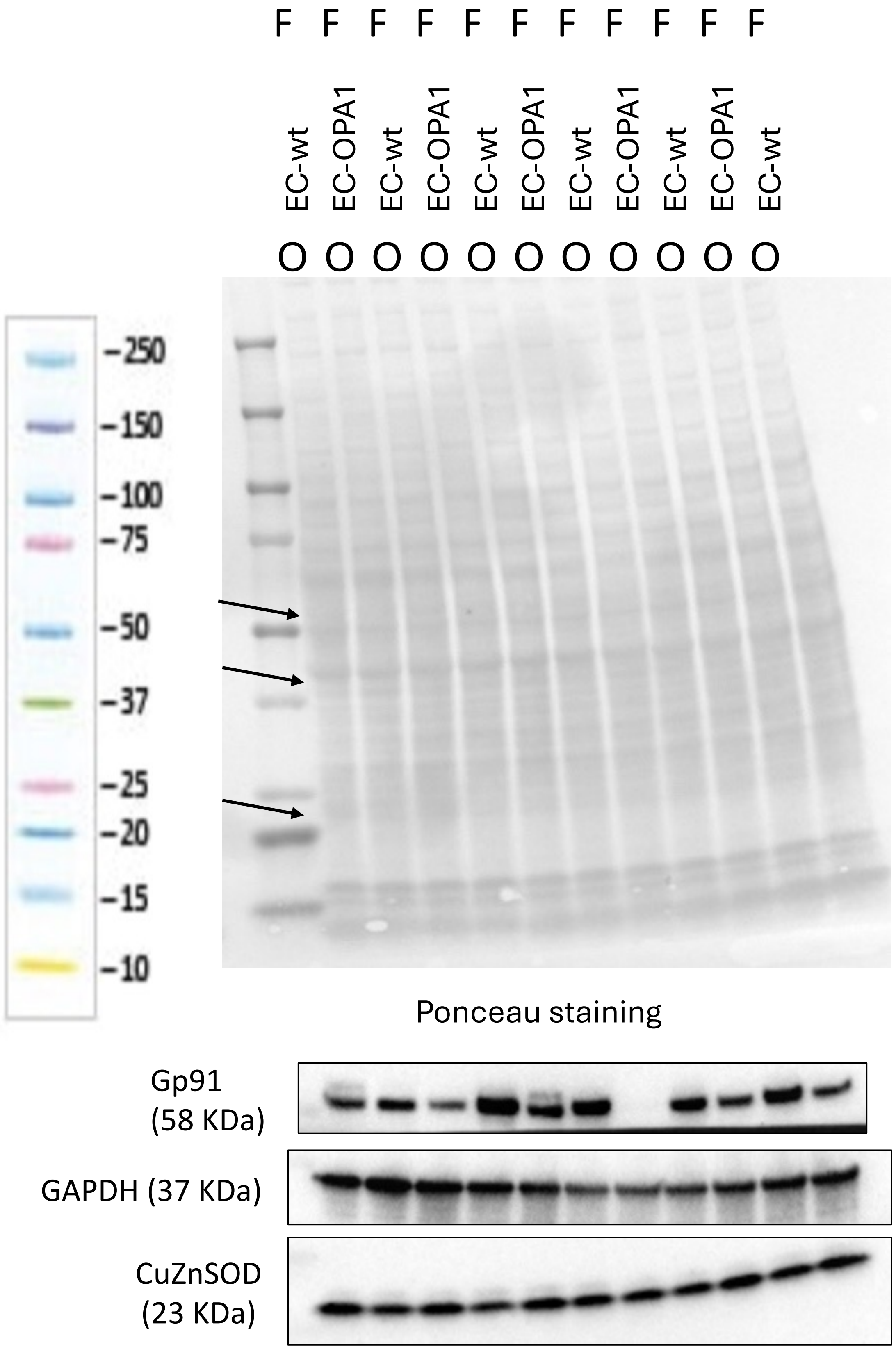

Legend: F = female, O = old

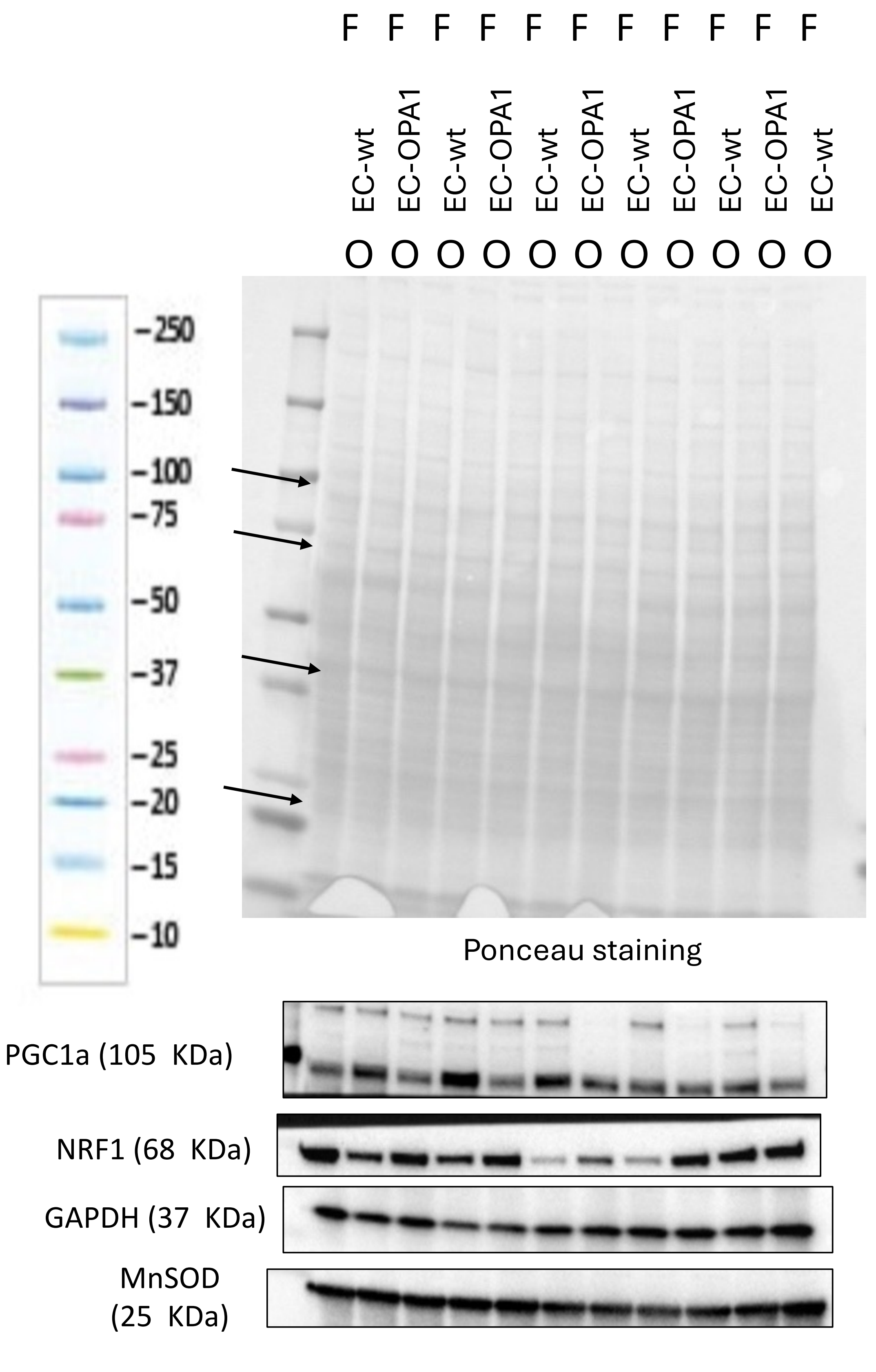

Legend: F = female, O = old

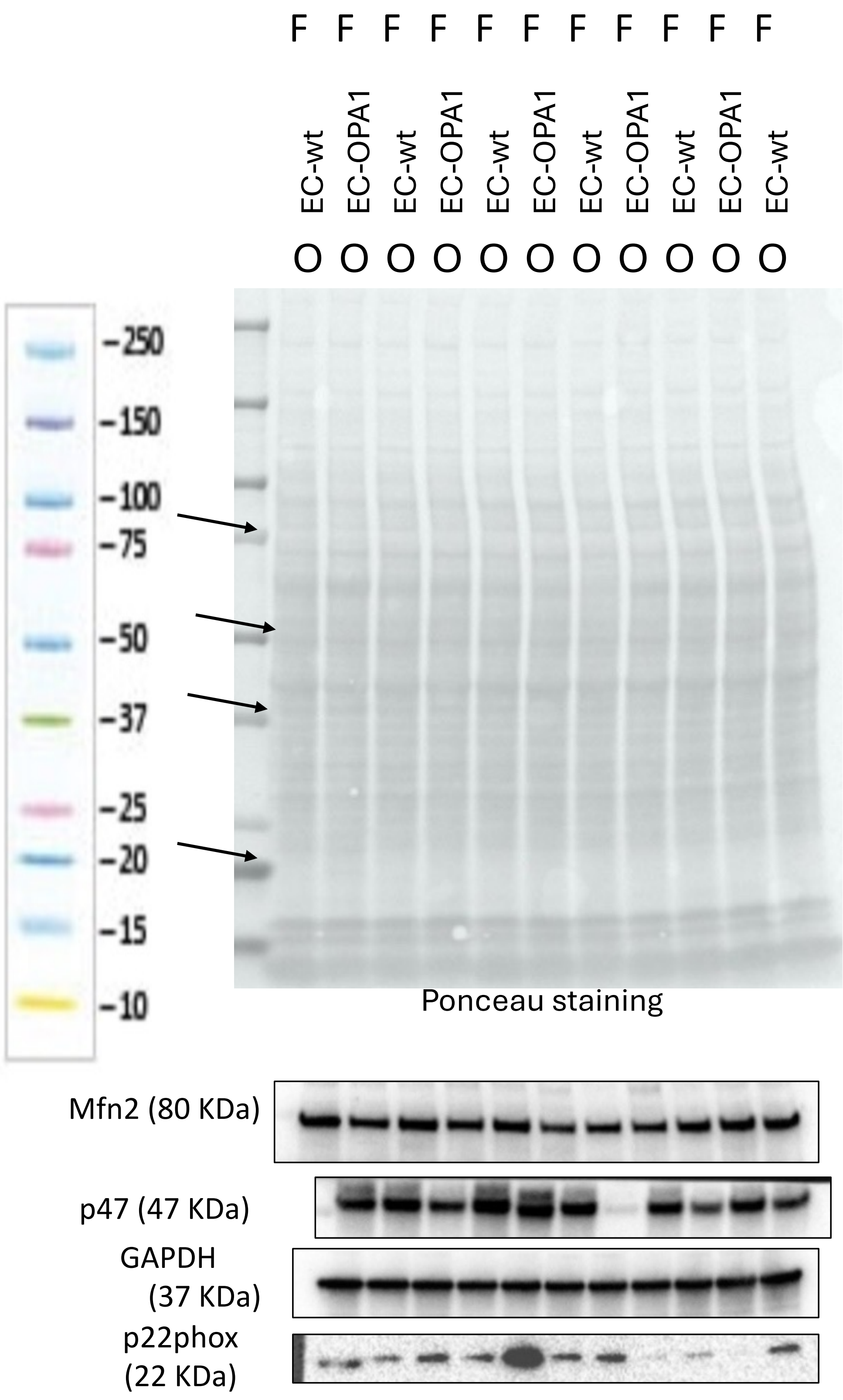

Legend: F = female, O = old

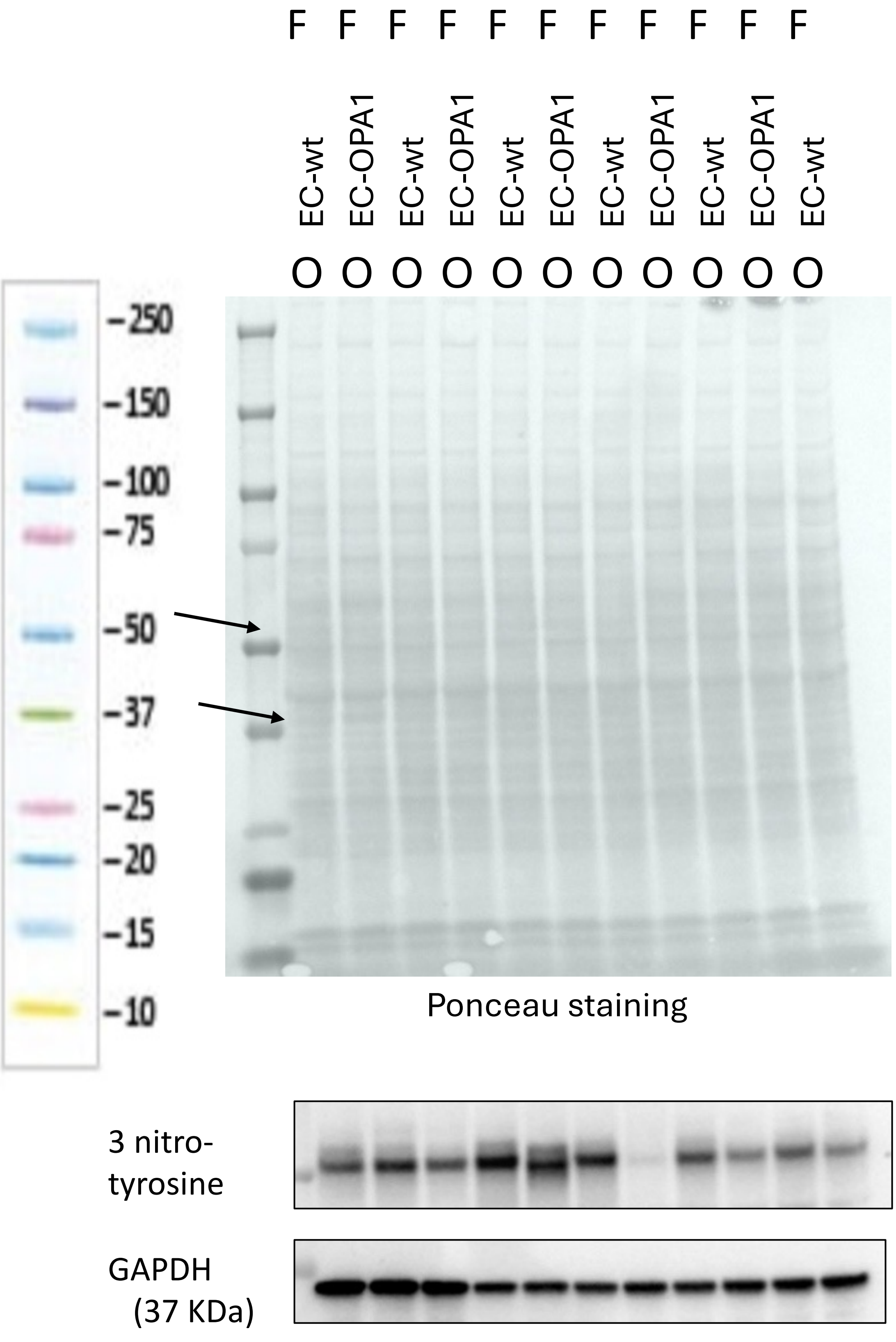

Legend: F = female, O = old

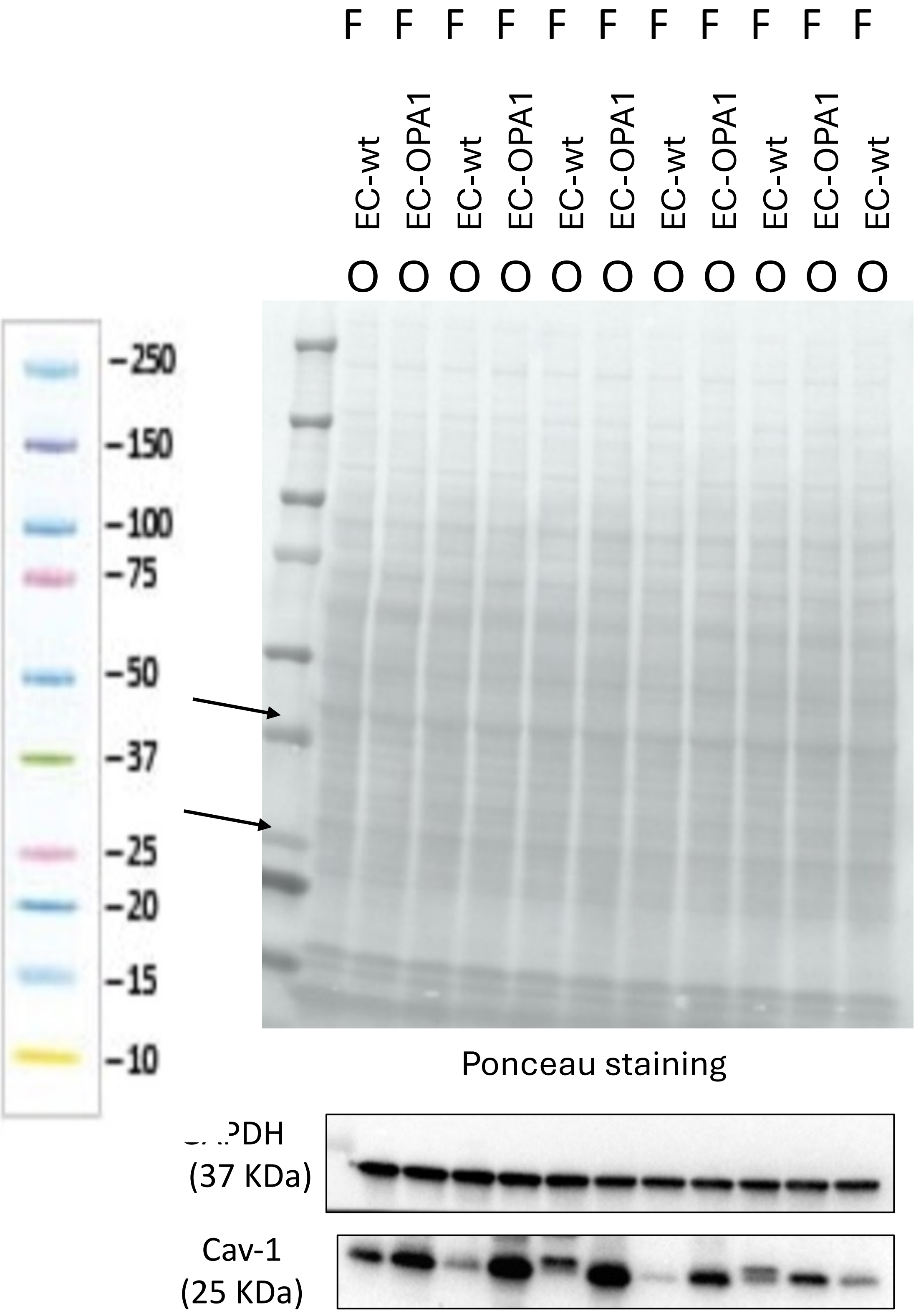

Legend: F = female, O = old

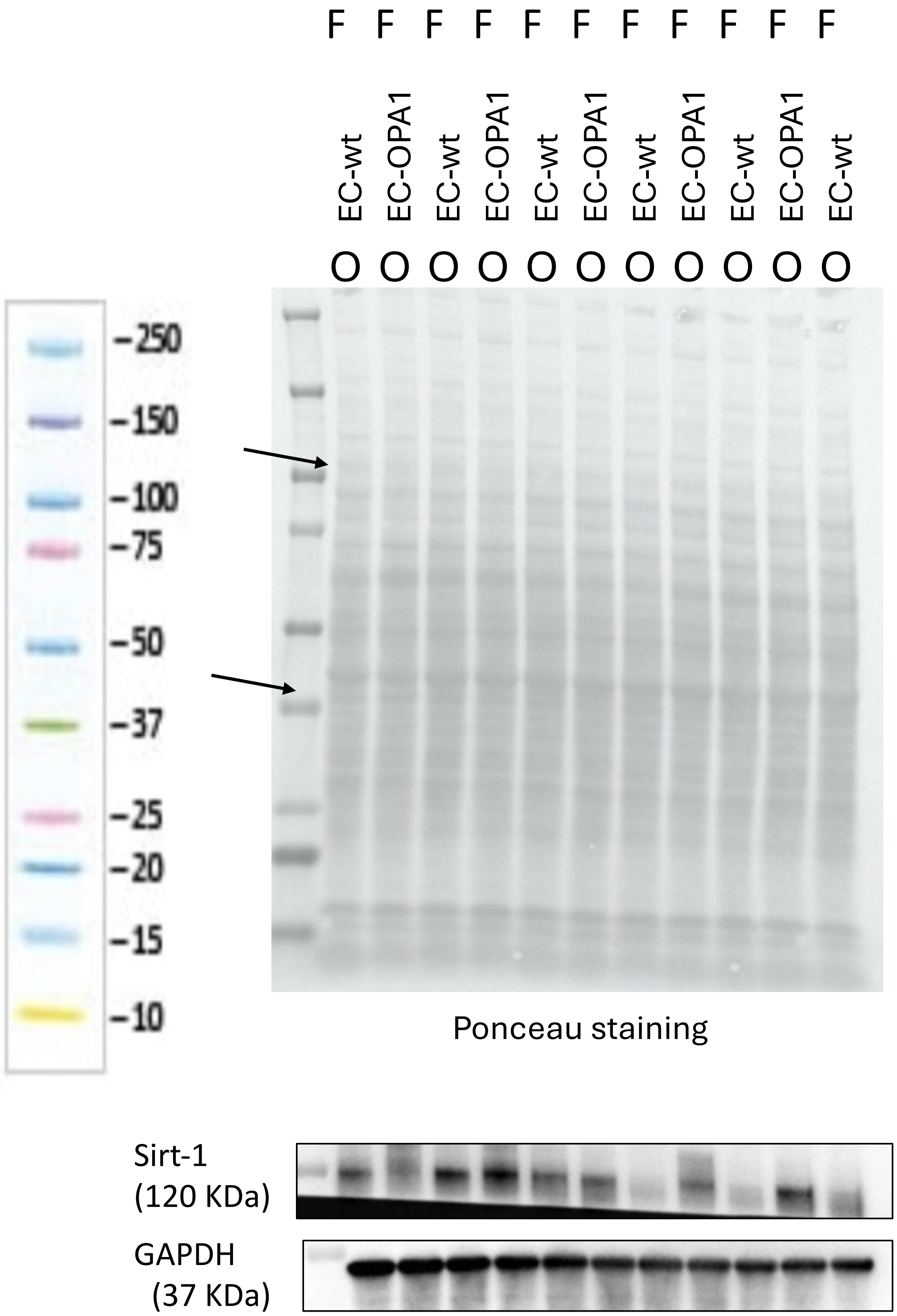

Legend: F = female, O = old

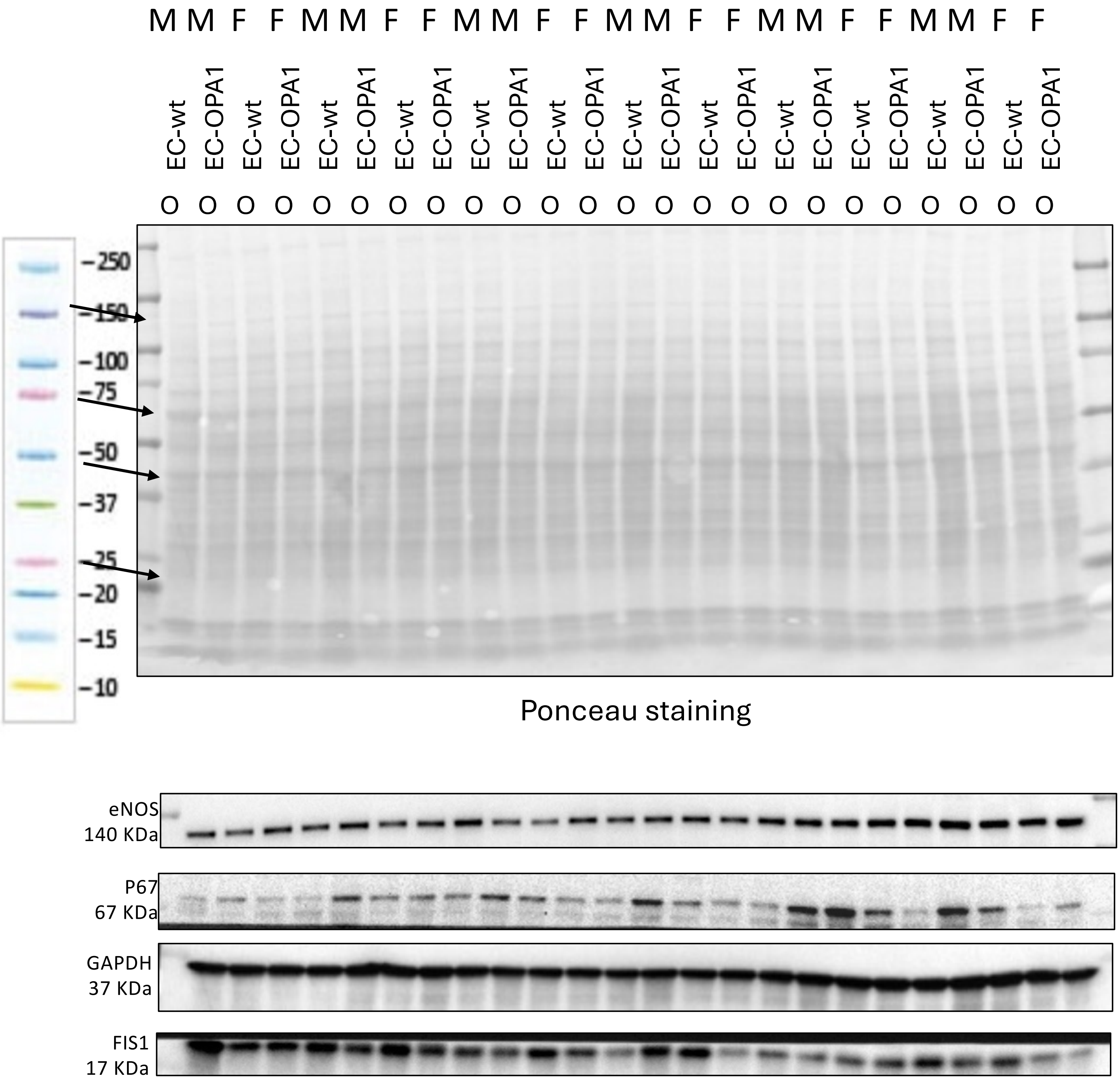

Legend: F = female, M = male, O = old

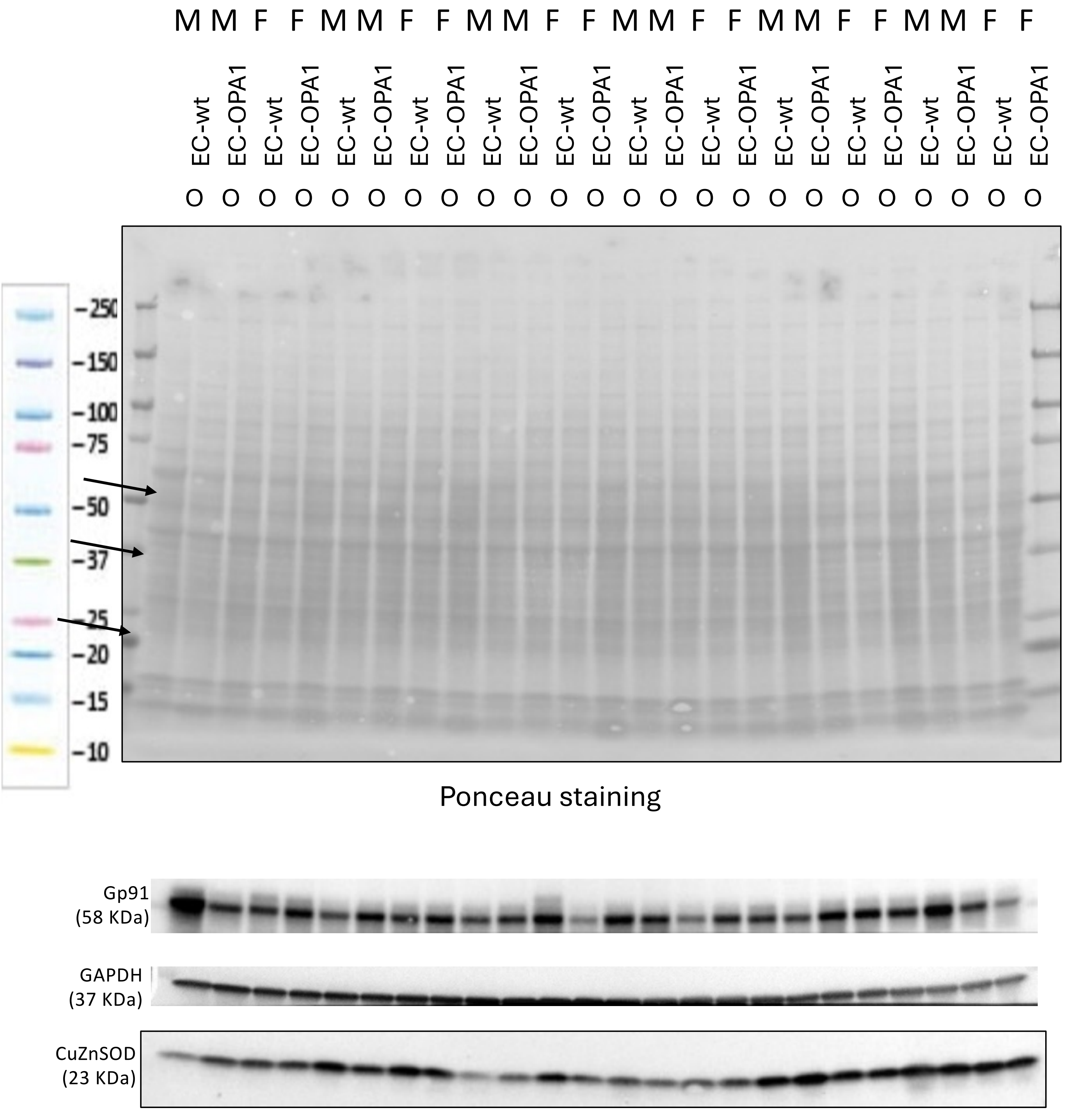

Legend: F = female, M = male, O = old

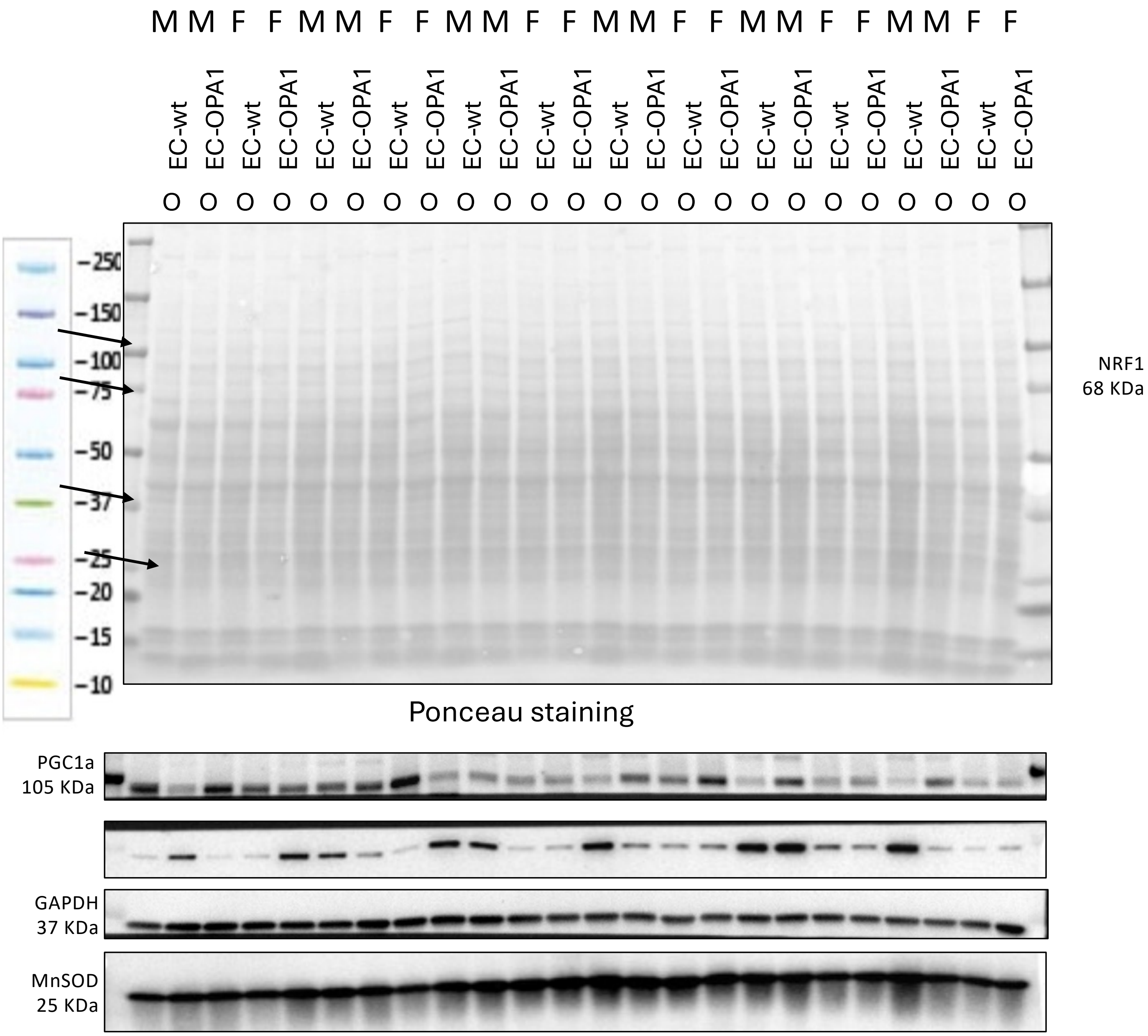

Legend: F = female, M = male, O = old

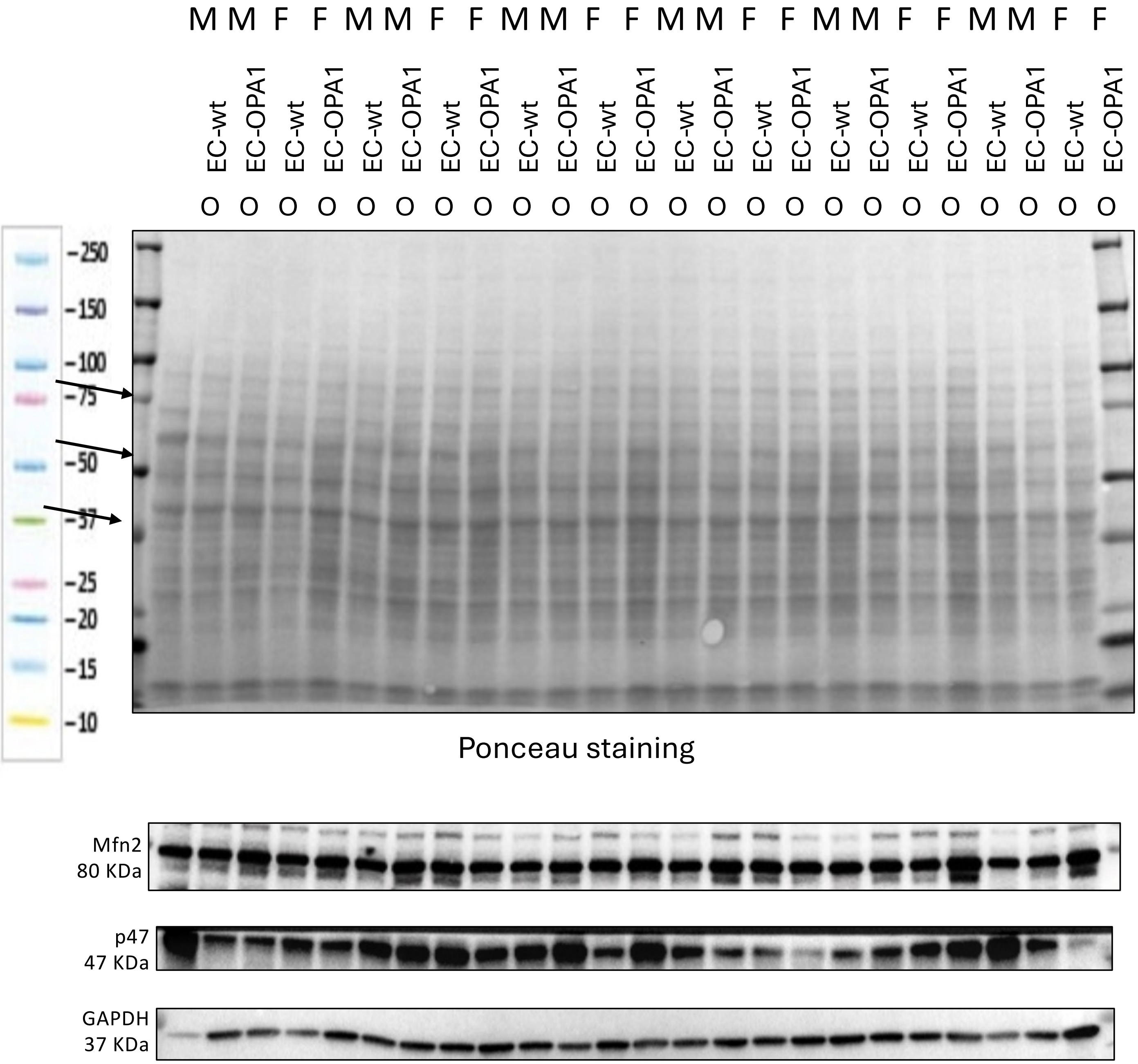

Legend: F = female, M = male, O = old

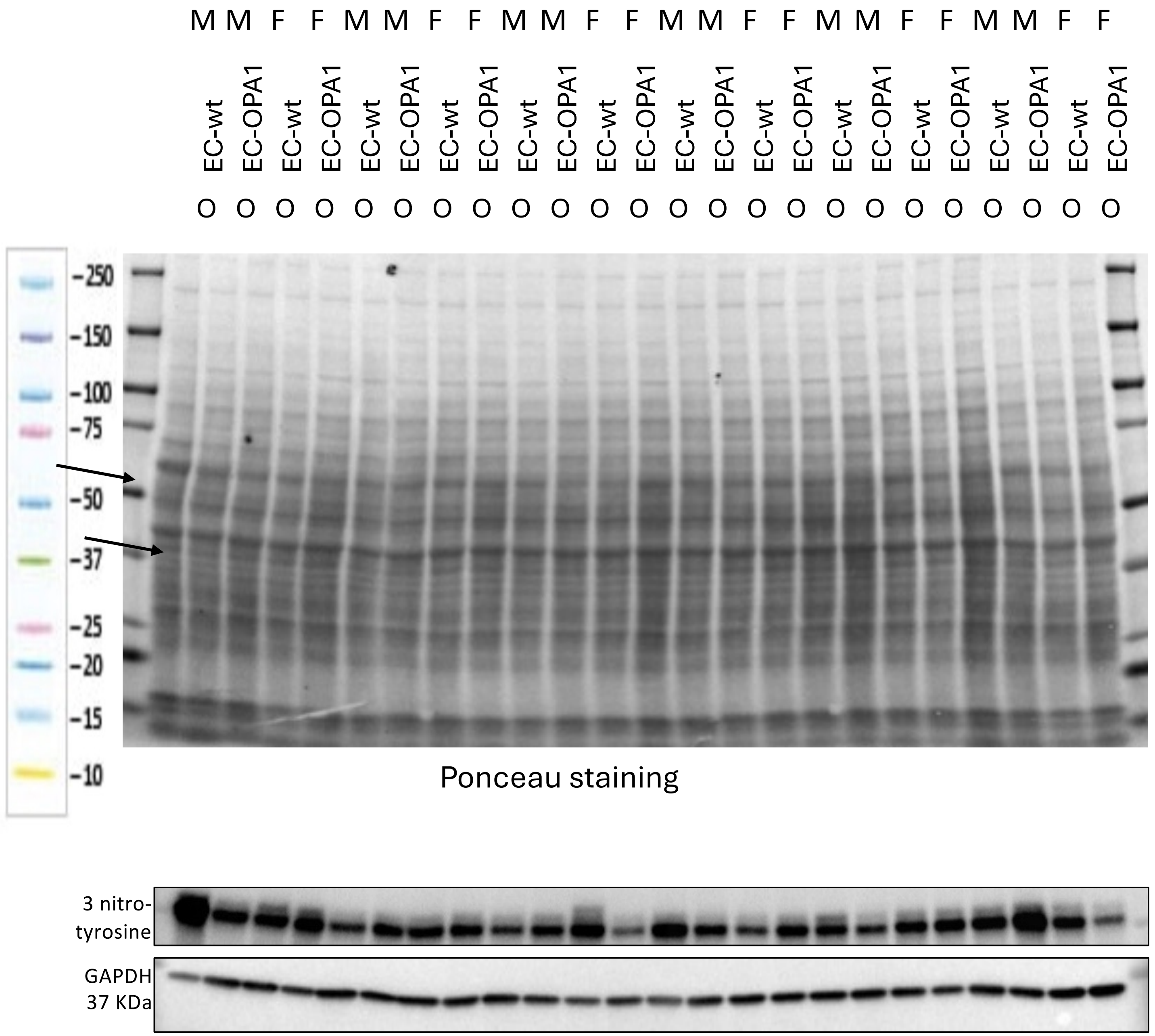

Legend: F = female, M = male, O = old

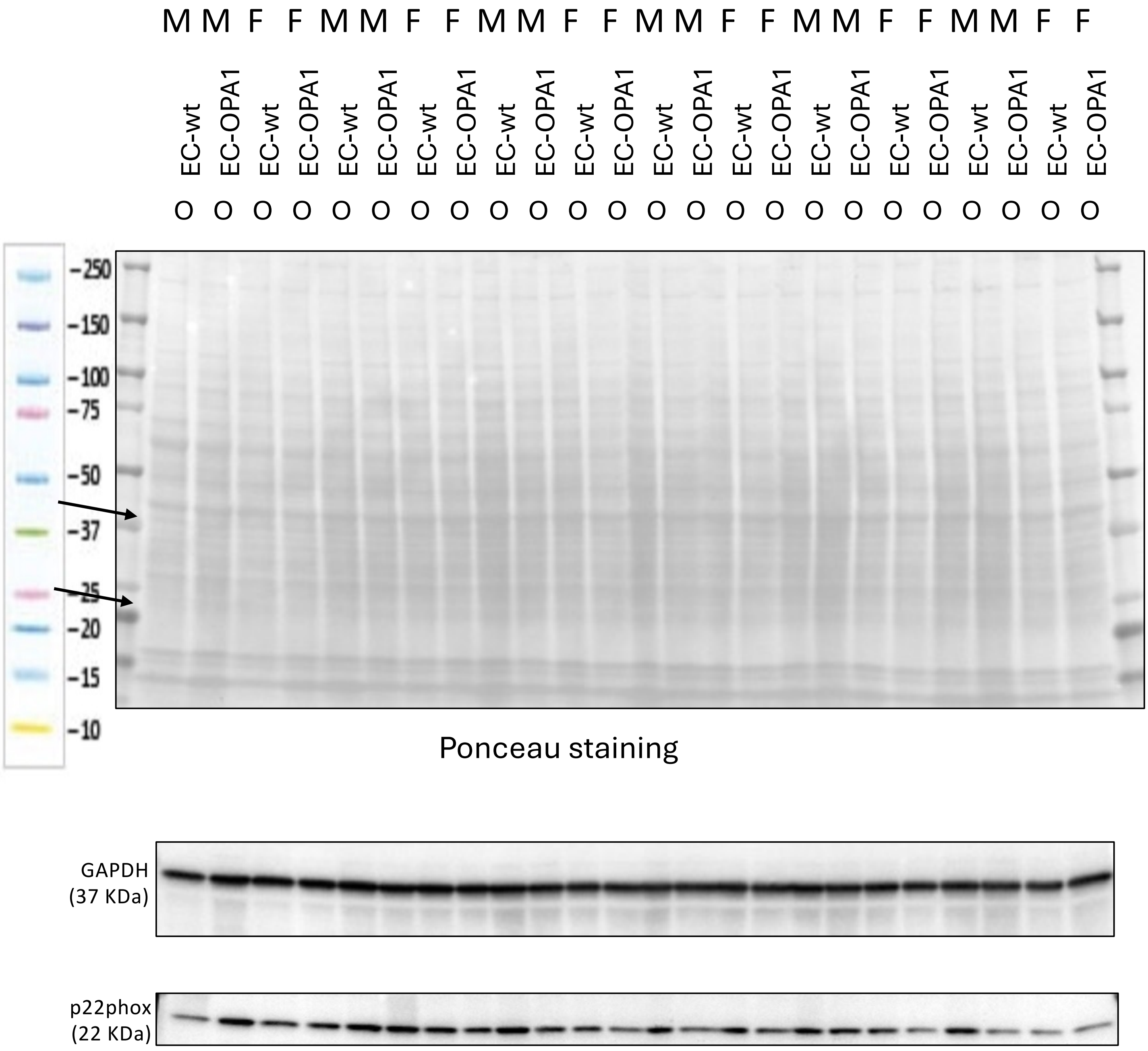

Legend: F = female, M = male, O = old

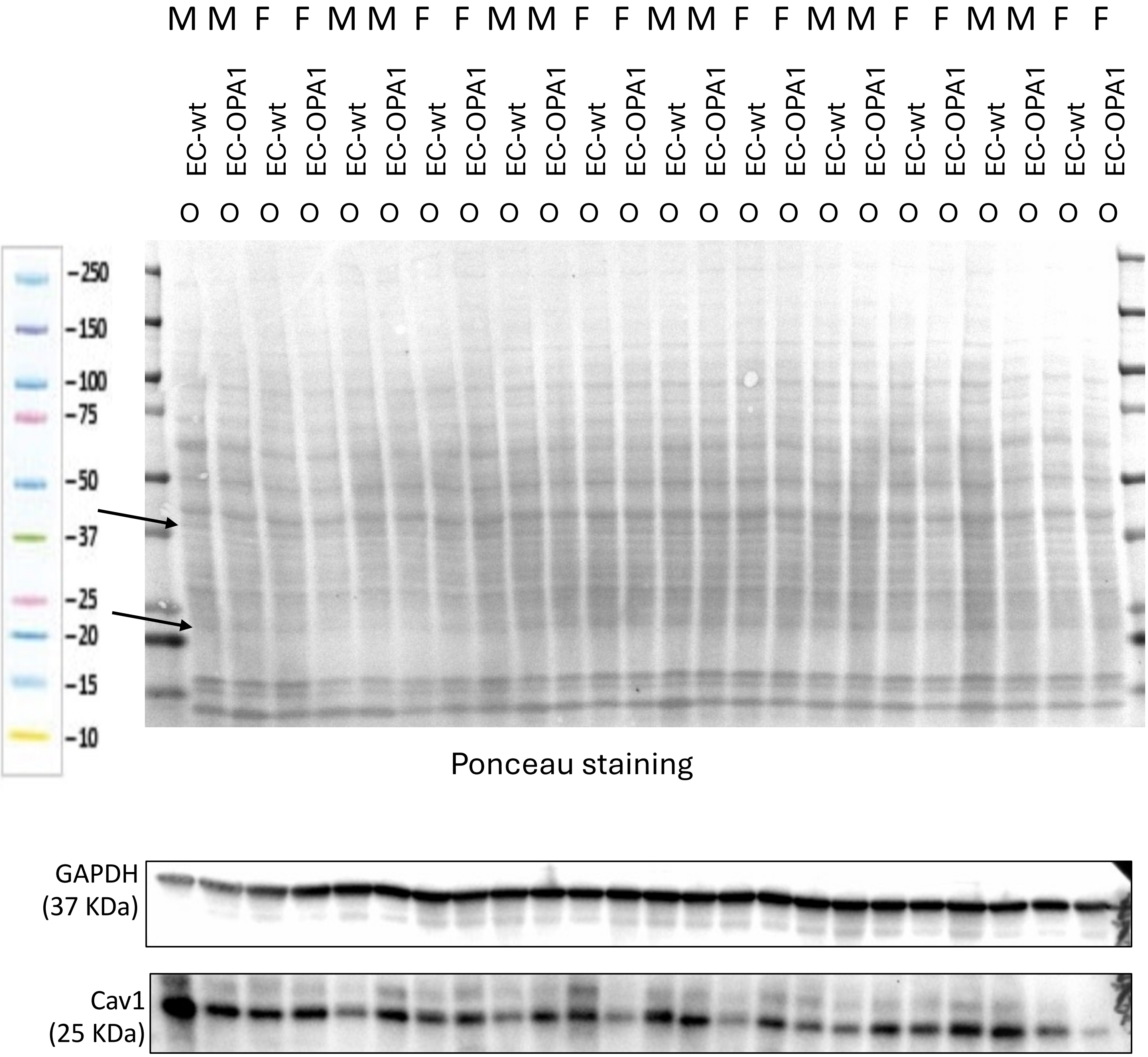

Legend: F = female, M = male, O = old

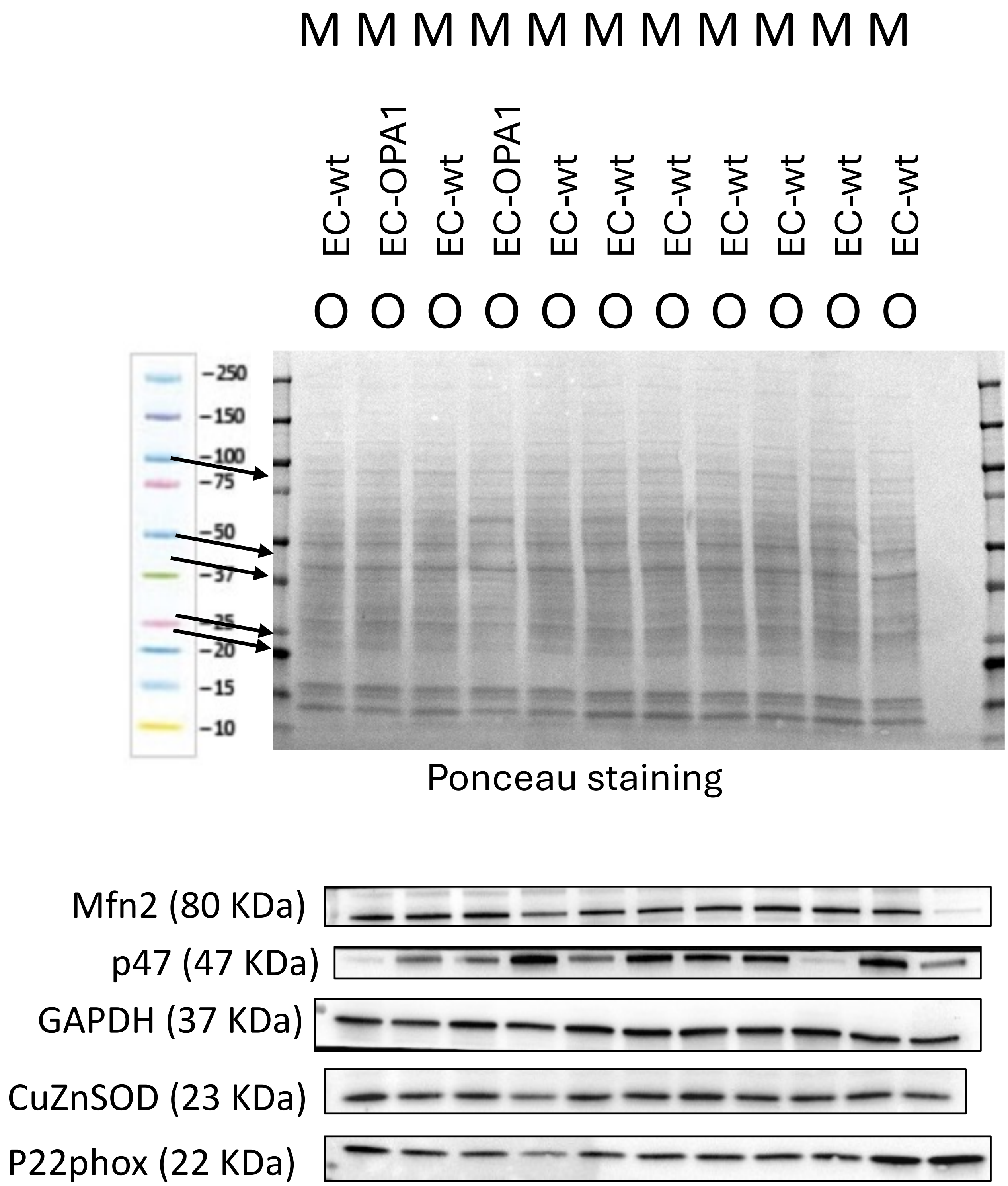

Legend: M = male, O = old

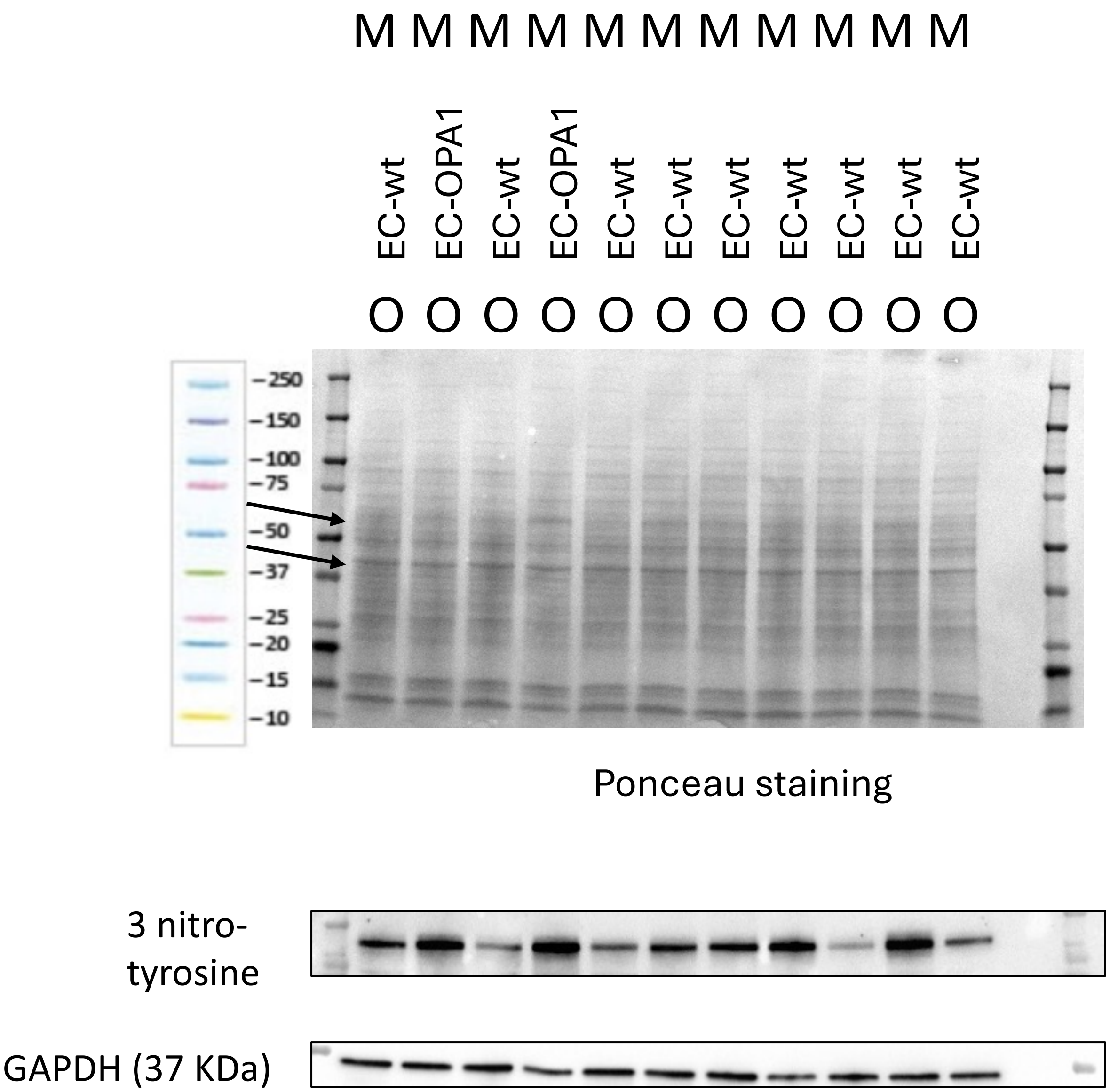

Legend: M = male, O = old

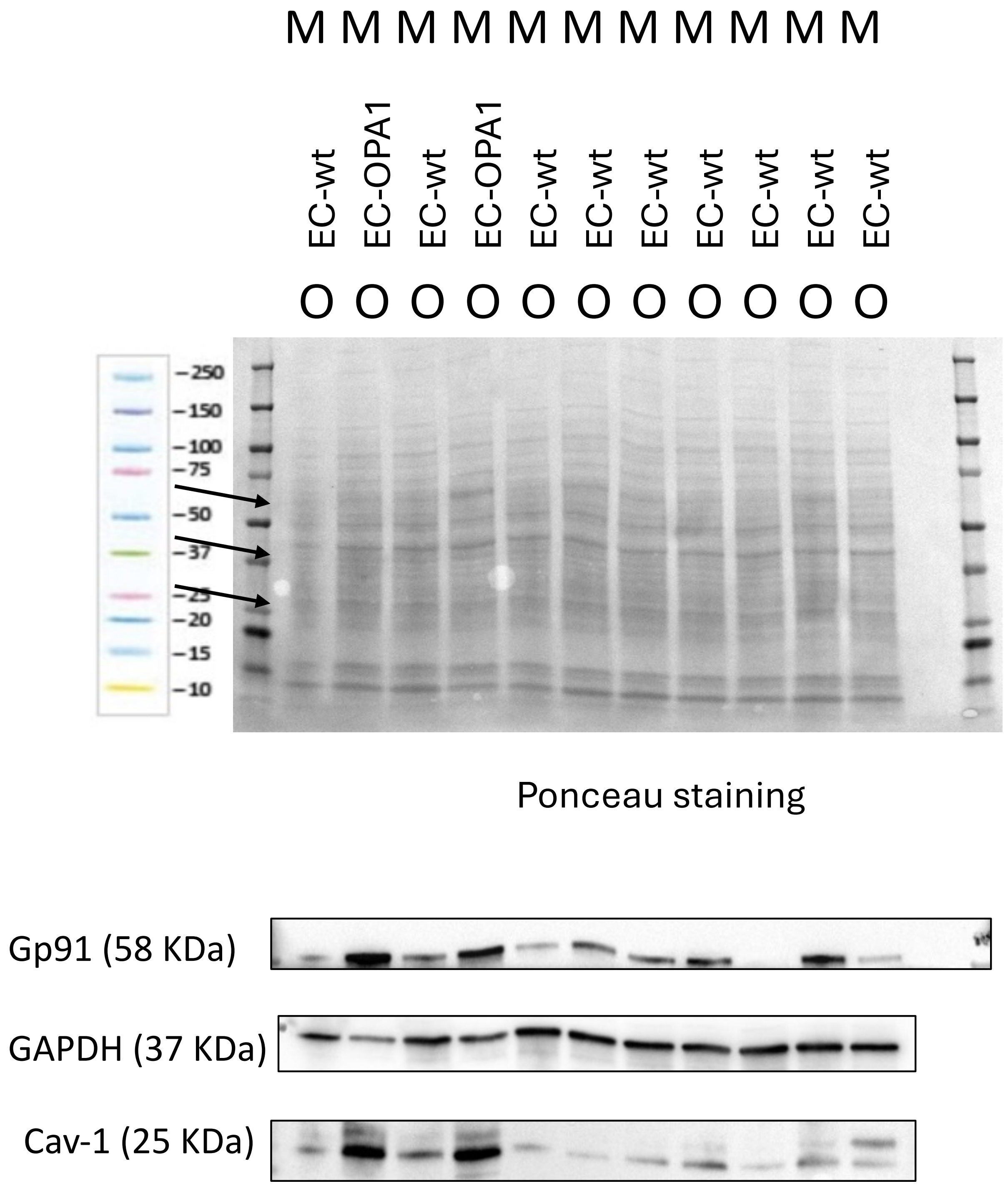

Legend: M = male, O = old

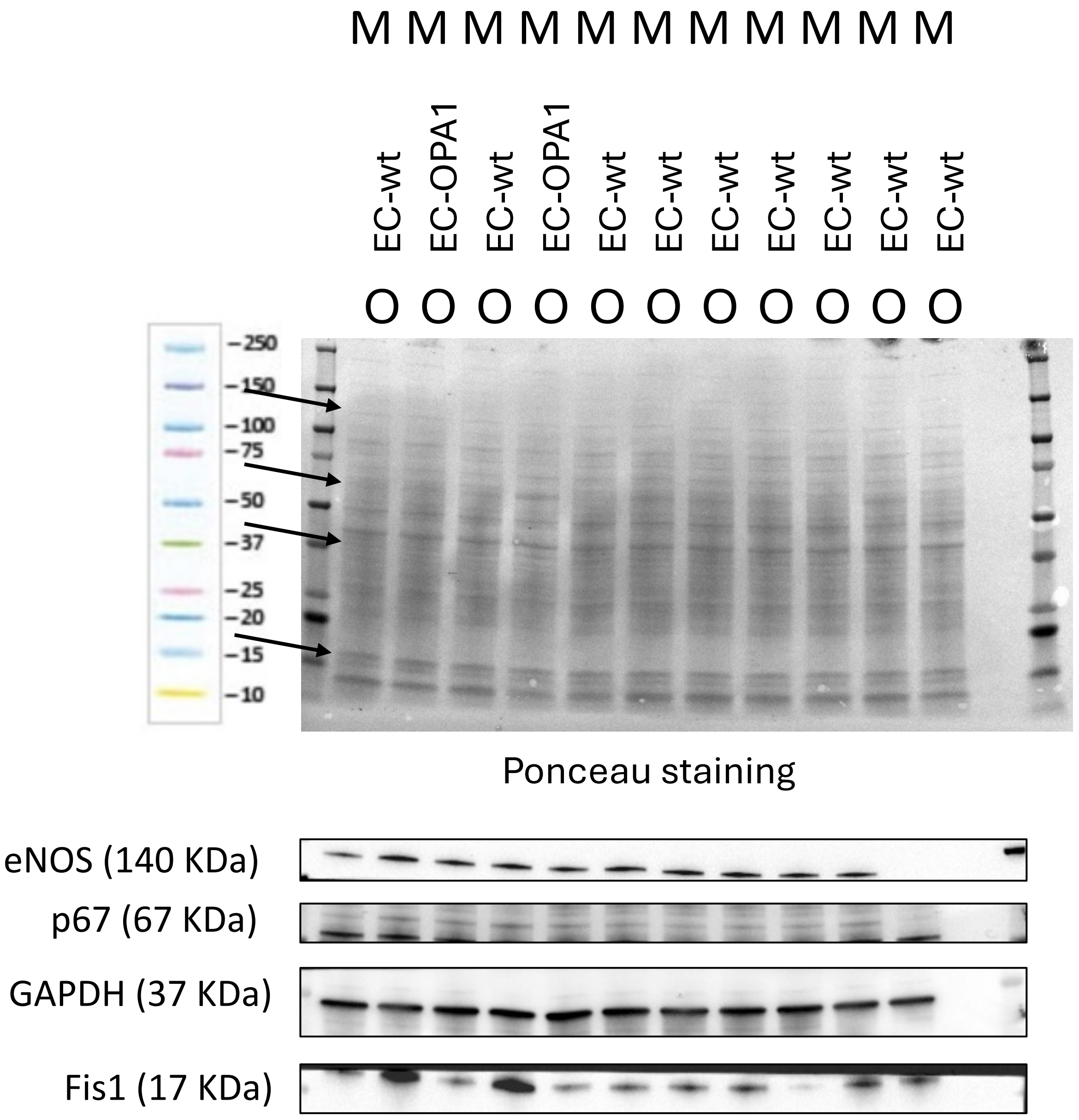

Legend: M = male, O = old

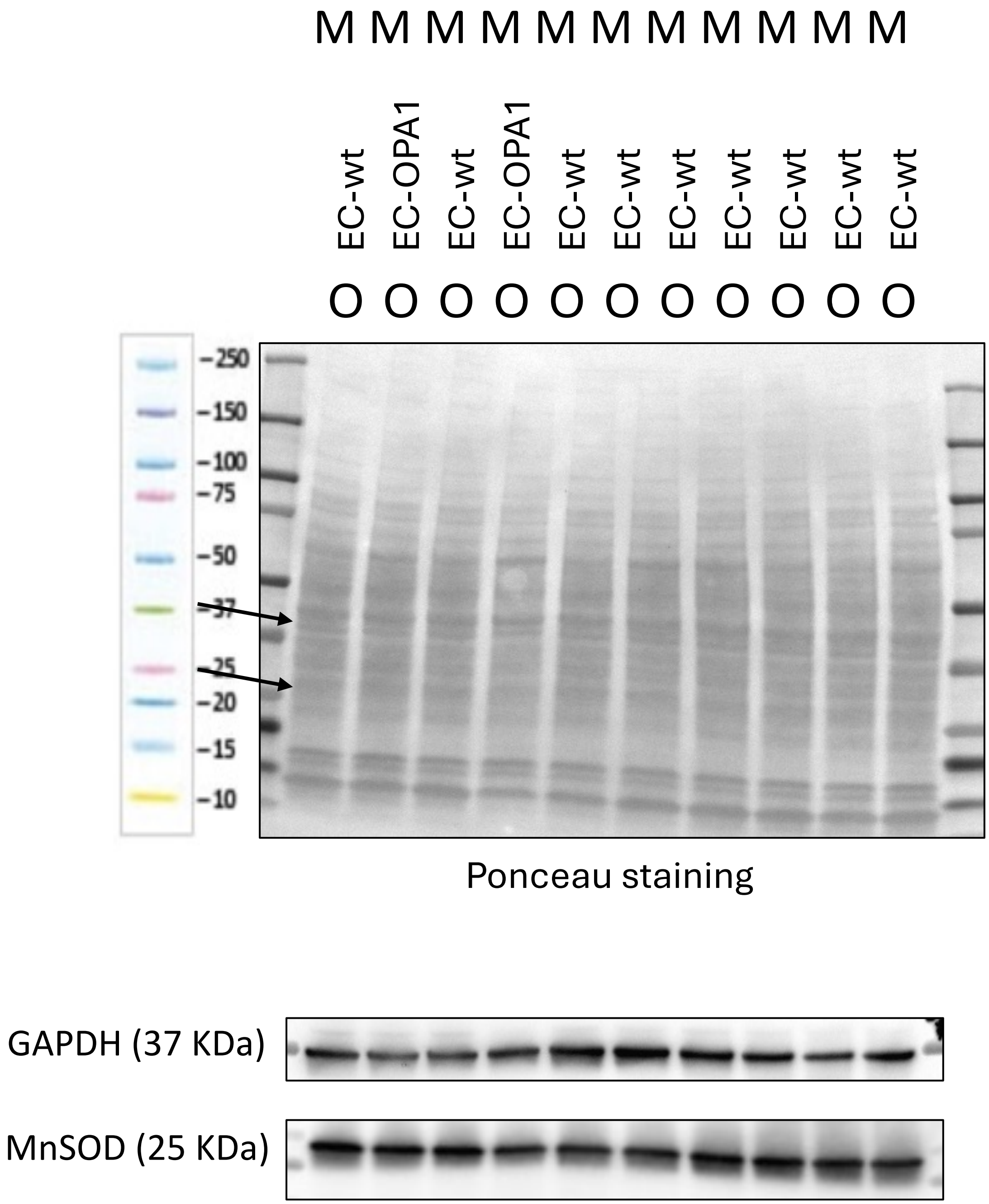

Legend: M = male, O = old

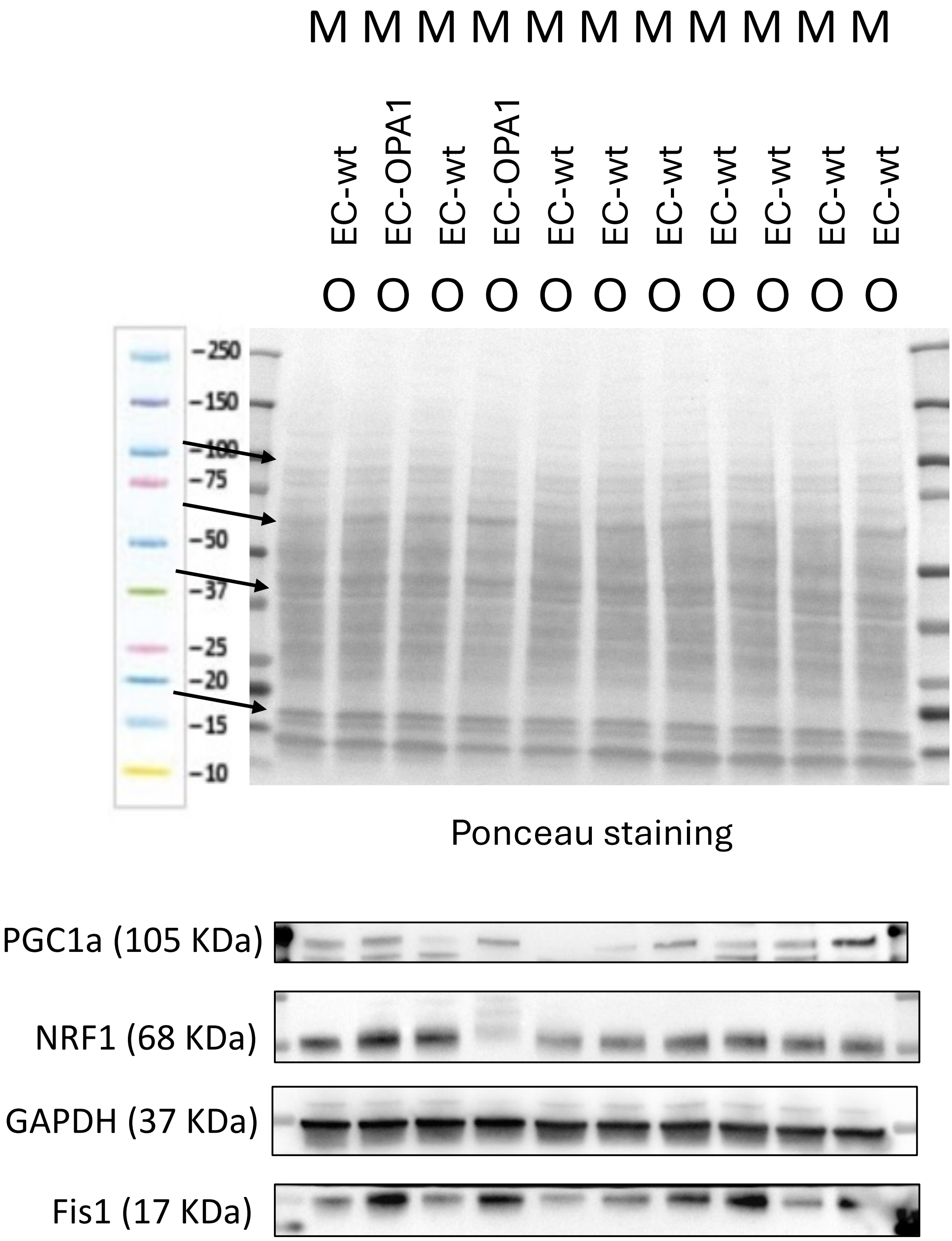

Legend: M = male, O = old
